# Presenteeism among healthcare professionals in a hospital setting: a scoping review

**DOI:** 10.1590/1518-8345.8286.4900

**Published:** 2026-08-10

**Authors:** Cynthia Lima Sampaio, Thailane Cardoso Soares, Victoria Lohreine de Carvalho Grangense, Bruna Victória da Silva Passos, Roberta Meneses Oliveira, Joselany Áfio Caetano

**Affiliations:** 1 Universidade Federal do Ceará, Departamento de Enfermagem, Fortaleza, CE, Brazil.

**Keywords:** Hospitals, Presenteeism, Occupational Health, Health Management, Health Personnel, Scoping Review

## Abstract

**Objective:**

to map the prevalence of presenteeism among healthcare professionals in the hospital environment, identify related factors, consequences for patient safety and worker health, as well as the interventions used.

**Method:**

scoping review based on the recommendations of the JBI Reviewer’s Manual, including studies available in full in five databases, with no time or language restrictions.

**Results:**

31 selected articles showed a high prevalence of presenteeism among healthcare professionals in the hospital setting, and the main related factors were illnesses (depression and infections), occupational aspects (work overload), and organizational aspects (lack of support from colleagues). The consequences for worker health were worsening health, stress, fatigue/exhaustion, and impairment of work capacity, while for patient safety, there was an increased risk of infections and clinical errors. Few studies have reported interventions, such as occupational wellness centers. The consequences also impacted economic aspects with loss of productivity and higher healthcare costs.

**Conclusion:**

the prevalence of presenteeism among healthcare professionals in the hospital setting is high and constitutes a multifactorial phenomenon with negative consequences for individuals, organizations, and patients. The study includes a multidimensional analysis of presenteeism and can serve as a reference for establishing hospital organizational health policies and programs. Multidimensional analysis of presenteeism can contribute to the establishment of organizational health policies and programs in hospitals. Given the scarcity of intervention studies, it is necessary to advance knowledge on the subject The research protocol is available on the Open Science Framework platform: https://doi.org/10.17605/OSF.IO/GVNX3.

## Highlights

**(1)** Presenteeism is a prevalent and multifactorial phenomenon among healthcare professionals in hospital settings. **(2)** Presenteeism contributes to the worsening of chronic diseases, stress, fatigue, and reduced work capacity. **(3)** In the organizational context, it compromises productivity, increases direct and indirect healthcare costs. **(4)** Negatively impacts patient safety, with a higher risk of errors and the spread of infections. **(5)** Intervention studies are needed to advance knowledge on this topic.

## Introduction

Healthcare institutions are recognized as extraordinarily complex work environments with high exposure to risks, including those familiar to other sectors as well as those inherent to healthcare practices. Healthcare professionals, especially those working in hospitals, are exposed to a wide variety of risks, including biological, chemical, physical, ergonomic, organizational, and psychosocial^1^.

In this context, absenteeism and presenteeism are common outcomes of these exposures and affect the productivity and well-being of professionals. However, while absenteeism among nursing professionals is widely studied due to its high cost to health systems, presenteeism is a more complex phenomenon that is difficult to measure because it involves multiple factors^2^.

The high costs of absenteeism can be illustrated by the annual loss of approximately US$ 4 million to the Eastern Province of Saudi Arabia^2^. Regarding presenteeism, existing studies exhibit substantial methodological heterogeneity and contradictory results regarding associations with occupational or organizational variables. This makes it difficult to draw definitive conclusions in the field of health^3^.

Furthermore, presenteeism is still a concept under development, but it is already recognized as the presence of professionals at work even when there are physical and mental limitations that prevent them from performing their work activities, although there is also a classification of presenteeism unrelated to illness (due to personal reasons, such as conflict between professional and personal life, perception of lack of organizational support, stress, etc.)^3^.

The post-COVID-19 pandemic scenario has increased interest in this phenomenon, as healthcare workers have faced exceptional emotional, physical, and organizational stress, with possible long-lasting repercussions for presenteeism, patient safety, and occupational health^4^. Recent studies point to high prevalence rates of presenteeism among hospital professionals, associated with exhaustion and repercussions on the quality of care and patient safety^4^ ^-^ ^5^.

Despite practical evidence on the impact of presenteeism on healthcare services, there is a lack of studies that integrate, in greater depth, prevalence, associated factors, consequences, and specific interventions in the hospital context.

This study is justified by the need for more in-depth research on the relationship between presenteeism, physical and mental exhaustion, burnout syndrome, stress, and depression, to enable effective interventions.

In the field of nursing, the relevance of this study lies in the fact that this category represents the largest workforce in hospital institutions and plays a significant role in direct patient care. Presenteeism among nursing professionals can affect the safety and quality of care and increase organizational costs.

Understanding presenteeism from a nursing management perspective is essential to support interventions that promote healthier work environments, reduce occupational risks, and encourage safe care practices.

Therefore, the objective was to map the prevalence of presenteeism among healthcare professionals in the hospital environment, identify related factors, assess the consequences for patient safety and worker health, and describe the interventions used.

## Method

### Type of study

The study is a scoping review that followed the steps recommended by the JBI Reviewer’s Manual, developed in five stages: formulation of the research question; identification of relevant studies; selection of studies; data extraction and analysis; synthesis and report construction. The Preferred Reporting Items for Systematic Reviews and Meta-Analyses Extension for Scoping Reviews (PRISMA-ScR) was used to write the study. The research protocol is registered on the Open Science Framework (OSF) platform under the Digital Object Identifier (DOI) https://doi.org/10.17605/OSF.IO/GVNX3. The search in the different databases was conducted in September 2024.

### Research question

The study question was developed using the acronym PCC, according to JBI recommendations. The population (P) was considered to be healthcare professionals; the concept of interest (C) was presenteeism; and the context (C) was the hospital; thus, formulating the following question: “What is the overview of presenteeism among healthcare professionals in the hospital environment?”

To answer the guiding question, other questions were asked: Q1. What is the prevalence of presenteeism among healthcare professionals in the hospital environment? Q2. What factors can influence presenteeism in this population? Q3. What are the consequences of presenteeism for patient safety and professional health in the hospital environment? Q4. What individual and organizational interventions are suggested to reduce presenteeism in the hospital environment?

### Selection criteria

The review covered full-length studies that answered the research question. The inclusion criteria were: publications without time or language restrictions, with full text available. Editorials, videos, websites, news articles, preprints, and abstracts were excluded.

### Search strategy

The search was conducted in the following databases: MEDLINE/PubMed (via the National Library of Medicine), Web of Science, CINAHL (via EBSCO), Scopus, and Embase, in the following steps: 1) initial search in MEDLINE to identify studies on the subject and select the words contained in these publications; 2) development of the search strategy; 3) validation of the search strategy developed with a librarian; 4) use of the new strategy.

The initial search strategy adopted was “presenteeism” OR “sickness presence” AND “hospitals” AND “healthcare workers.” After consulting with the librarian, the strategy was defined as follows, using MeSH (Medical Subject Headings): ((Presenteeism OR “Sickness Presence” OR “Presence, Sickness”) AND (Hospitals OR “Hospital Administration”) AND (“Health Personnel” OR “Personnel, Health” OR “Healthcare Workers” OR “Healthcare Worker” OR “Health Care Providers” OR “Health Care Provider” OR “Provider, Health Care” OR “Healthcare Providers” OR “Healthcare Provider” OR “Provider, Healthcare” OR “Health Care Professionals” OR “Health Care Professional” OR “Professional, Health Care”)) and as follows, using EMTREE: ((‘presenteism’ OR ‘sickness presence’ OR ‘working while ill’ OR ‘working while sick’ OR ‘presenteeism’) AND (‘health care practitioner’ OR ‘health care professional’ OR ‘health care provider’ OR ‘health care worker’ OR ‘health personnel’ OR ‘health profession personnel’ OR ‘health worker’ OR ‘healthcare personnel’ OR ‘healthcare practitioner’ OR ‘healthcare professional’ OR ‘healthcare provider’ OR ‘healthcare worker’ OR ‘home health aides’ OR ‘personnel, health’ OR ‘public health officer’ OR ‘health care personnel’) AND hospital).

The strategy with descriptors registered in EMTREE was used in the Embase database, and the strategy with descriptors registered in MeSH was used in the other selected databases. Access to the databases was provided through the Journal Portal of the Coordination for the Improvement of Higher Education Personnel (CAPES).

It should be noted that research was conducted on the International Prospective Register of Systematic Reviews (PROSPERO) and the OSF platforms to identify reviews on the study’s subject, but none were found.

A new search was conducted in November 2025 in the Brazilian Digital Library of Theses and Dissertations (BDTD) and in the CAPES thesis and dissertation catalogs, using the following strategy: *Presenteeism* AND *Hospitals* AND “*Health Personnel*.” Only one study was found, and it was repeated in both repositories. This study had already been included in the initial database search.

### Study selection

After searching the databases, all identified records were grouped and uploaded to the Rayyan (Intelligent Systematic Review) application^6^. In the first step, duplicates were removed. After removal, the titles and abstracts were read and evaluated by two independent reviewers, applying the inclusion and exclusion criteria. Potentially eligible studies were obtained in full text and evaluated by the same reviewers. Disagreements over which studies would comprise the sample were discussed among the reviewers, and when no consensus was reached, a third reviewer was called in.

The participating reviewers have training in nursing and are members of a Teaching, Research, and Extension Center in Health Management and Care at a federal university, in the field of research: Organizational health care and patient safety care and management. The screening and selection stage was initially conducted by two independent reviewers, one of whom was a doctoral student with experience in scope review and the other a member of the scientific initiation program. The first reviewer selected 31 studies, and the second, 15, agreeing on 12 records, for a 35% agreement. Given the observed divergence, a third reviewer, a master’s student with experience in scope review, conducted a new screening and selected 29 studies, all of which matched the first reviewer’s choices, resulting in total agreement between the two (kappa = 1.00). 

The reasons for exclusion in full text were recorded and presented in the PRISMA-ScR flowchart. 

### Instrument used for data collection

Data extraction was performed using an instrument developed by the authors, based on the form suggested by the JBI manual and protocol publications, and adapted to the study object^7^. The data extracted were: study identification (author, title, year, country, design/outline, objective); participants (total number, professional training, study location); results found (prevalence of presenteeism among healthcare workers in the hospital environment, related factors, consequences for patient safety and worker health, interventions used to reduce presenteeism); conclusions.

The information was organized in a Microsoft Excel^®^ spreadsheet, enabling control over the steps and cross-checking of data. No data were altered or deleted without the reviewers’ agreement.

### Data processing and analysis

Based on the extracted data, a descriptive analysis was performed, and tables and figures were constructed using data from the publications. The results of the research and the study inclusion process are reported in full in the final scope review and presented in a Preferred Reporting Items for Systematic Reviews and Meta-analyses extension for scoping review (PRISMA-ScR) flowchart.

The synthesis of results was organized narratively, grouping findings according to conceptual and methodological similarities. We chose not to perform a critical assessment of methodological quality, in line with JBI recommendations for scoping reviews.

The factors influencing presenteeism were identified in the studies using statistical tests of association and correlation, including: chi-square (χ²), Student’s t-test, and ANOVA for associations, and Pearson’s correlation (r) and Spearman’s correlation to measure correlations^8^.

### Ethical aspects

This study did not involve human subjects, and the data used are considered secondary data in the public domain with free access. In addition, all references regarding copyright^9^ were included. Therefore, there was no need to submit the study to the Ethics and Research Committee.

## Results

**Figure 1:**
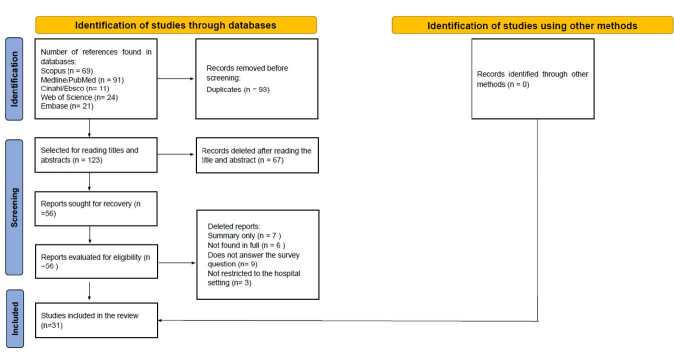
PRISMA flowchart used for study identification and selection. Fortaleza, CE, Brazil, 2025

Source: PRISMA flowchart for study selection

The searches yielded 216 records. After excluding duplicates, 123 remained for selection. Upon analysis of the titles and abstracts, 56 were selected; and after reading the studies in full, 31 were included in the review (Figure 1).

The 31 studies show wide diversity in terms of country of origin, year of publication, methodological design, and instruments used (Figure 2). Most are cross-sectional studies (n = 22; 70.97%), followed by three prospective longitudinal studies (9.68%) and three retrospective studies (9.68%). In addition, one methodological study (3.23%), one systematic review (3.23%), and one letter to the editor (3.23%) were identified.

**Figure 2:** Identification of studies included in the sample according to code, country, year of publication, design, questions answered in the review, and instruments used. Fortaleza, CE, Brazil, 2025

Article code	Country/Year	Design	Answers to Review Questions (P1 to P4)	Measuring instruments
A1^10^	Switzerland, 2021	Longitudinal prospective	P1	Charlson Comorbidity Index History of flu vaccination and household characteristics that could affect the risk of flu Respiratory symptom diaries and mask-wearing adherence
A2^11^	Nigeria, 2021	Cross- sectional	P1 and P2	Oldenburg Burnout Inventory (OLBI) Stanford Presenteeism Scale (SPS-6) Patient Health Questionnaire-2 (PHQ-2)
A3^12^	China, 2021	Cross-sectional	P2	Perceived Ability to Work Scale (PAWS) Challenge and Hindrance-related Self-Reported Stress Scale (C-HSS) Short Form Health Survey (SF-8) Public Service Motivation Scale
A4^13^	China, 2019	Cross-sectional	P1 and P2	Perceived Ability to Work Scale (PAWS) Short Form Health Survey (SF-8) Challenge and Hindrance-related Self-Reported Stress Scale (C-HSS) Short-form PSM Scale Job in General Scale Turnover Intention Scale Job Performance Scale *Chirurgisches Qualitätssiegel Scale*
A5^14^	China, 2022	Cross-sectional	P1 and P2	Niehoff & Moorman Scale (distributive justice). Pierce, et al. Scale (organizational self-esteem) Perceived Ability to Work Scale (PAWS) Eisenberger, et al. Scale (Perceived Organizational Support)
A6^15^	Turkey, 2024	Methodological	P2	Stanford Presenteeism Scale (STAS) Happiness At Work Scale Burnout Scale Short Version
A7^16^	China, 2024	Cross-sectional	P2	Impact of Events Scale (IES) Turnover Intention Scale (PTSD) Stanford presenteeism Scale (STAS) Social Support Scale (SSS)
A8^17^	Brazil, 2021	Cross-sectional	P1 and P2	Stanford Presenteeism Scale (STAS) - Brazilian version Safety Attitudes Questionnaire (SAQ)
A9^18^	Malta, 2020	Cross-sectional	P2 and P3	Questionnaire on perceptions of illness during episodes of presenteeism and absenteeism Brief Illness Perception Questionnaire (B-IPQ)
A10^19^	France, 2022	Systematic review	P3	Stanford Presenteeism Scale (SPS-6) Work Limitations Questionnaire (WLQ) Work Productivity and Activity Impairment (WPAI) Health and Work Performance Questionnaire (HPQ) Perceived Ability to Work Scale (PAWS)
A11^20^	Brazil, 2022	Cross-sectional	P1, P2 and P3	Stanford Presenteeism Scale (SPS-6) Work Ability Index (WAI)
A12^21^	Japan, 2020	Letter to the editor	P1	Two open-ended questions about symptoms, recognition of illness, and reasons for coming to work sick.
A13^22^	United Kingdom, 2024	Cross-sectional	P4	Warwick-Edinburgh Mental Wellbeing Scale (WEMWBS) Four global measures of single-item stress at work, job satisfaction, presenteeism, and turnover intentions.
A14^23^	United Kingdom, 2020	Cross-sectional	P1 and P4	Warwick-Edinburgh Mental Wellbeing Scale (WEMWBS) Global single-item scales (stress, satisfaction, intention to leave, and presenteeism). Utrecht Work Engagement Scale (UWES - dedication subscale, 3 items) Open questions about perception, benefits, and barriers of the centers
A15^24^	Australia, 2021	Cross-sectional	P1 and P2	Question about how many times they went to work when they were sick or not feeling well in the last 12 months.
A16^25^	China, 2017	Cross-sectional	P1 and P2	Perceived Ability to Work Scale (PAWS)
A17^26^	United States, 2017	Cross-sectional	P1	Anonymous online self-report questionnaire
A18^27^	Portugal, 2016	Longitudinal prospective	P1 and P3	Work Limitations Questionnaire8 (WLQ8) (8-item short form) Stanford Presenteeism Scale (SPS6)
A19^28^	China, 2019	Cross-sectional	P1 and P2	Perceived Ability to Work Scale (PAWS) Short Form Health Survey (SF-8) Challenge and Hindrance-related Self-Reported Stress Scale (C-HSS) Short-form PSM scale Ironson, et al. Scale (job satisfaction) Singh scale, et al. (intention to rotate) Darwish Scale (work performance) *Chirurgisches Qualitätssiegel* Scale
A20^29^	Australia, 2020	Longitudinal retrospective	P1	Presenteeism inferred from the absence of sick leave records during the period of confirmed infection Absenteeism measured by hours of leave recorded / total hours scheduled × 100 Laboratory, administrative, and payroll data integrated via employee identification number.
A21^30^	China, 2019	Cross-sectional	P2	Perceived Ability to Work Scale (PAWS)
A22^31^	Japan, 2022	Longitudinal retrospective	P3	No questionnaire or standardized presenteeism scale was used.
A23^32^	Croatia, 2017	Cross-sectional	P1 and P2	Patient Safety Culture. Health and Work Performance Questionnaire (HPQ)
A24^33^	China, 2018	Cross-sectional	P1 and P2	Perceived Ability to Work Scale (PAWS) Challenge and HindranceRelated SelfReported Stress Scale
A25^34^	Slovenia, 2020	Cross-sectional	P1 and P3	Questionnaire developed by the authors themselves, with several questions about health, work, and behavior
A26^35^	Spain, 2018	Cross-sectional	P1 and P2	Stanford Presenteeism Scale6 (SPS-6)
A27^36^	Japan, 2020	Cross-sectional	P1 and P2	Questionnaire developed by the author for demographic variables and symptoms Work Productivity and Activity Impairment - General Health (WPAIGH)
A28^37^	Thailand, 2022	Longitudinal prospective	P1	Direct recording by observers during the shift and questionnaire with self-reported symptoms in the last seven days
A29^38^	Israel, 2024	Longitudinal prospective	P1	Participant reports, combined with active surveillance data
A30^39^	Iran, 2022	Cross-sectional	P1 and P2	Valuation of Lost Productivity Questionnaire (VOLP), adapted for COVID-19 WHO STEPwise
A3^40^	United States, 2019	Cross-sectional	P1 and P2	Anonymous questionnaire developed by the authors with closed-ended items on reasons for presenteeism

As for countries, studies conducted in China predominated (n = 8; 25.81%), followed by Japan (n = 3; 9.68%), Australia (n = 2; 6.46%), Brazil (n = 2; 6.45%), the United Kingdom (n = 2; 6.45%), the United States (n = 2; 6.45%), and one study for Switzerland, Nigeria, Malta, Turkey, France, Portugal, Croatia, Slovenia, Spain, Thailand, Israel, and Iran (3.23%). Regarding the year of publication, there was a higher concentration in 2020 (n = 6; 19.35%) and 2022 (n = 6; 19.35%), followed by 2021 (n = 5; 16.13%), 2019 (n = 4; 12.90%), and 2024 (n = 4; 12.90%). The other years had lower frequencies: 2017 (n = 3; 9.68%), 2018 (n = 2; 6.45%), and 2016 (n = 1; 3.23%).

The instruments used to assess presenteeism included standardized scales such as the Perceived Ability to Work Scale (PAWS) (n=8), Stanford Presenteeism Scale (SPS-6) (n=7), Work Limitations Questionnaire (WLQ) (n=2), Work Productivity and Activity Impairment (WPAI) (n=2), Health and Work Performance Questionnaire (HPQ) (n=2), and Work Limitations Questionnaire (WLQ) (n=2). (n=7), Work Limitations Questionnaire (WLQ) (n=2), Work Productivity and Activity Impairment (WPAI) (n=2), Health and Work Performance Questionnaire (HPQ) (n=2), and Valuation of Lost Productivity Questionnaire (VOLP), adapted for COVID-19 (n=1). In addition to these, some studies used their own questionnaires containing direct questions about the frequency of presenteeism behavior and other instruments to measure associated constructs, such as Burnout, Motivation for work, Happiness at work, Social Support, Change Stress, Turnover, among others (n= 26).

Of the thirty-one articles selected for the sample, twenty-three answered the question about the result of presenteeism measurement (P1), as shown in Figure 3. The studies analyze presenteeism from different perspectives, such as working while sick or loss of productivity, and use various scales. Some studies provide data on the prevalence of presenteeism and the intensity with which it occurs. Others disclose only the prevalence, average, or unit of measurement (loss of productivity, degree, or financial value).

**Figure 3:** Studies on the prevalence of presenteeism among hospital professionals by country, year, number of participants, instruments used and main results. Fortaleza, CE, Brazil, 2025

Stanford Presenteeism Scale (SPS-6)			
Country, year	Number of participants	Results about presenteeism	
		Prevalence	Mean
Nigeria, 2021^11^	155	unreported	22.1 (SD* 5.1)
Spain, 2018^35^	323	52.9%	19.84 (SD* 4.2)
Brazil, 2021^17^	754	43.5%	20.9 (SD* 4.7)
Brazil, 2022^20^ ^)^	299	65.6%	21.36 (SD* 4.7)
**Perceived Ability to Work Scale (PAWS)**			
China, 2017^25^	1392	unreported	7.25 (SD* 1.79) a 7.63 (SD* 1.57)
China, 2019^13^	572	unreported	3.92 (SD* 2.33) a 4.12 (SD* 2.38)
China, 2019^28^	1434	unreported	7.44 (SD* 1.716) a 7.71 (SD* 1.59)
China, 2018^33^	1392	unreported	2.37 (SD* 1.67) a 2.75 (SD* 1.79)
China, 2022^14^	1122	unreported	2.09 (SD* 1.46) a 2.35 (SD* 1.61)
**Work Limitations Questionnaire- 8 (WLQ-8) e SPS-6**			
Portugal, 2016^27^	188	unreported	WLQ-8: Loss of 19.56% productivity per working day. SPS-6: nurses 1.95 (0.96) and assistants 2.28 (0.70)
**Work Productivity and Activity Impairment-General Health (WPAI-GH)**			
Japan, 2020^36^	668	62.1%	The degree of presenteeism was 30.
**Valuation of Lost Productivity Questionnaire (VOLP) adapted for COVID-19**			
Iran, 2022^39^	250	100%	US$ 1,489.05 (SD* = US$ 1,143.15) within a period of three months.
**Health and Work Performance Questionnaire (WHO-HPQ)**			
Croatia, 2017^32^	595	70%	6.82%
**Questions about perceptions of presenteeism developed by the author**			
Japan, 2020^21^	90	Prevalence of 48.9%	
United Kingdom, 2020^23^	819	Prevalence of 68.6%	
Australia, 2021^24^	320	Prevalence of 87.8%	
United States, 2017^26^	286	Prevalence of 92.0%	
Israel, 2024^38^	2505	Prevalence of 72.3% (IC^†^ de 95%: 60.4-86.4).	
Slovenia, 2020^34^	5865	Prevalence of 57.0%	
Thailand, 2022^37^	240	Prevalence of 23.0%	
**Analysis of laboratory-confirmed influenza cases**			
Australia. 2020^29^	1540	Prevalence of 14.1%	
Switzerland. 2021^10^	152	Prevalence of 67.9%	
**Other scales**			
Japan. 2020^40^	14	Prevalence of 64.0%	

*SD = Standard deviation; ^†^CI = Confidence interval

The results show wide variation in the prevalence of presenteeism, ranging from 14.1% in Australia to 100% in Iran. Regarding the scale averages, variations ranging from 2.37 (SD = 1.67) to 7.71 (SD = 1.59) were observed in the PAWS, and from 19.84 (SD = 4.2) to 22.1 (SD = 5.1) in the SPS-6.

Only three studies compared presenteeism among different professional categories. In one of them, nurses had lower levels of presenteeism (14.48%) than operational assistants (27.19%)^27^. In another, presenteeism was higher among physicians^39^. A Brazilian study found lower productivity loss among physicians than among all professionals in the sample (23.85 points), followed by nursing technicians and nurses, who scored 20.91 points and 20.65 points, respectively, indicating a lower reduction in their productivity in the face of health problems^20^.

Nineteen articles addressed factors that may influence presenteeism (P2). These data were found in the articles through associations and correlations (Figure 4). A study with a direct question about the reason for working while sick found a higher percentage (56%) of responses for “sense of duty as a health professional”^40^.

**Figure 4:** Factors that may influence presenteeism verified through associations and correlations in the studies reviewed. Fortaleza, CE, Brazil, 2025

Significant associations found between presenteeism and factors identified in the reviewed studies
**Demographic factors:** 🚬Smoking (Smokers with higher presenteeism: OR* = 2.99. p = 0.030)^(39)^ 👴Age (Middle age: OR* = 11.189, p = 0.046)^(39)^ 👴Age (30 years old or younger, p = 0.023)^(15)^ 💍Single (p = 0.014)^(15)^ 🏘Living alone (p = 0.030)^(15)^ 🚻 Gender (Women at 1.88 times greater risk), IC^†^: 1.06-3.32, p = 0.03)^(20)^ 🚻Gender (Men) (p < 0.05)^(13)^
**Health-disease factors:** 🤯Stress (p < 0.01)^(35)^ 😔Depression (p < 0.015)^(11)^ 💪Musculoskeletal disorders (p < 0.05)^(18)^ ❤Chronic diseases (p < 0.05)^(18)^ 🤕Headaches/migraines (p < 0.05)^(18)^ 🦠COVID-19 (one infection: OR* = 6.816, p = 0.000), two infections: OR* = 24.023. p = 0.001)^(39)^
**Occupational factors:** 🏥Hospital emergency (p = 0.004)^(35)^ 📊Negative safety climate (p < 0.05)^(17)^ 💊Patient safety culture^(32)^ 📈Workload and career length (mid-career/end-of-career professionals)^(13)^ 📈Working 9 hours or more daily (p < 0.001)^(15)^ 📈5 years or less in the profession (p = 0.012)^(15)^ 💰 Income (Third and fourth quartiles with the highest presenteeism)^(39)^ 🩺Physician (p = 0.005)^(15)^ ⚖️Fairness in the distribution of rewards and recognition (e.g., salaries, workload, promotions) has higher levels of presenteeism^(14)^
**Significant correlations were found between presenteeism and factors identified in the reviewed studies**
**Workload** Exhaustion (-0.350. p < 0.001)^‡^ ^(11)^ Disengagement (-0.475. p < 0.001)^‡^ ^(11)^ Excessive working hours (r = 0.105. p = 0.016)^§^ ^(36)^ Shift work (r = 0.106. p = 0.015)^§^ ^(36)^ Workaholism (r = 0.232. p < 0.001)^§^ ^(36)^
**Organizational commitment** Organizational commitment (β = −0.42, p < 0.001)^‡^ ^(28)^ Support from coworkers (β = −0.15, p < 0.001)^‡^ ^(28)^ Motivation in public service (β = −0.35; p < 0.001)^‡^ ^(30)^ and (β = −0.13, p < 0.01)^‡^ ^(12)^ Affective commitment (β = −0.27; p < 0.001)^‡^ ^(25)^ Happiness at work (r = -0.567. p < 0.001)^‡^ ^(15)^
**Health-illness** Challenge stress (β = 0.07, p < 0.01)^§^ ^(12)^ Challenge stress (-0.05, p = 0.001)^‡^ ^(33)^ Impediment stress (β = 0.13, p < 0.01)^§^ ^(12)^ and (0.25, p = 0.001)^§^ ^(33)^ Health (β = −0.12, p < 0.01)^‡^ ^(12)^ and (r = -0.35)^‡^ ^(33)^ Challenge stress and impedance stress (r = 0.20, r = 0.32)^§^ ^(25)^ PTSD^||^ (β = 0.638, p < 0.001)^§^ ^(16)^ Intensity of low back pain (r = 0.248, p < 0.001)^§^ ^(36)^. Sleep duration (-0.131, p = 0.003)^‡^ ^(36)^ Depressive symptoms (r = 0.269, p < 0.001)^§^ ^(36)^ Emotional stability (r = -0.226, p ≤ 0.01)^‡^ ^(24)^ Work-related stress (r= 0.218, p ≤ 0.001)^§^ ^(24)^ Burnout (r= 0.653, p < 0.001)^§^ ^(15)^

*OR = Odds Ratio; ^†^CI = Confidence interval; ^‡^Negative correlation; ^§^Positive correlation; ^||^PTSD = Post-traumatic stress disorder

The association factors identified were divided into: demographic, health-illness, and occupational, revealing the importance of age, gender, smoking, physical and mental illnesses, income, sector, workload, and safety climate.

Factors such as exhaustion, disengagement, organizational commitment, support from coworkers, motivation in public service, emotional commitment, happiness at work, better overall health, longer sleep duration, and emotional stability were associated with lower presenteeism.

On the other hand, longer working hours, shift work, challenge and impediment stress, depressive symptoms, greater intensity of low back pain, and the presence of PTSD (Post-traumatic stress disorder) symptoms were correlated with higher presenteeism.

**Figure 5:**
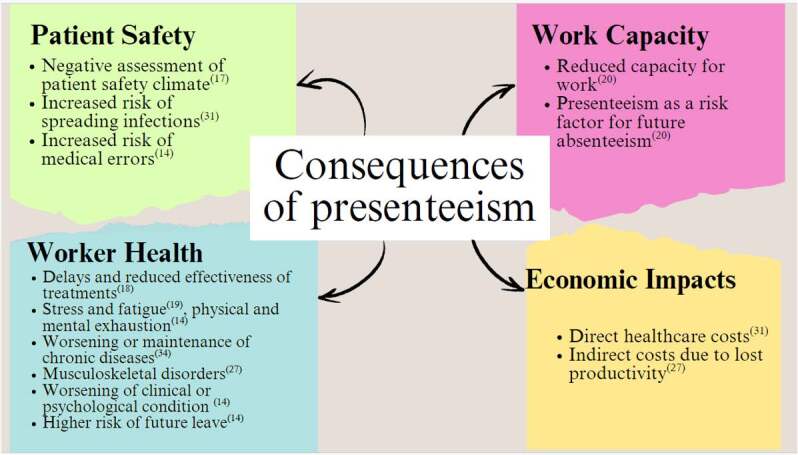
Mind map on the consequences of presenteeism. Fortaleza, CE, Brazil, 2025

Regarding the consequences of presenteeism (P3), seven articles were found that addressed the repercussions for patient and worker safety, as well as the impact on work and economic capacity, as shown in Figure 5.

Regarding the individual and organizational interventions adopted (P4), only 2 studies reported the use of wellness centers as an institutional strategy. In one of these investigations, it was observed that healthcare professionals without access to wellness centers had higher levels of presenteeism (β = −0.22; p < 0.001) and were, on average, younger (β = 0.09; p = 0.002). In addition, a significant interaction was identified between presenteeism and access to the wellness center on professionals’ well-being levels, with *F* (1, 791) = 18.65; *p* < 0.001; ηp² = 0.02. Professionals who presented presenteeism and accessed the center reported higher levels of well-being (mean = 3.30; standard deviation - SD = 0.04) than those who accessed the center but did not report presenteeism (mean = 3.06; SD = 0.040)^22^.

On the other hand, a second study did not identify statistically significant differences between professionals who accessed wellness centers and those who did not in terms of the following variables: perception of work stress, job satisfaction, presenteeism, or intention to turnover^23^.

## Discussion

The results presented provide a comprehensive overview of various aspects of presenteeism. The guiding questions about the prevalence of presenteeism and the factors that influence it were largely answered through the mapping carried out. However, answers to questions about consequences and interventions were found in only a few studies.

The findings reinforce the heterogeneity of instruments for measuring presenteeism and the importance of considering multiple contextual and methodological factors when interpreting the results. Regarding the prevalence of evaluated presenteeism, results vary depending on the measurement instrument used.

PAWS was the most widely used scale for investigations into productivity loss, followed by SPS-6. The original instrument assesses self-perception of work capacity, with scores ranging from 0 to 10, where higher scores indicate greater work capacity and better performance. In the studies included in this review, the authors invert the scores for a more intuitive interpretation, where higher scores indicate greater presenteeism, while lower scores reflect lower presenteeism or greater perceived work capacity. The SPS-6, on the other hand, allows the influence of health problems on work quality and professional performance to be measured, proving to be an easy-to-understand tool that facilitates its completion and analysis^15^. 

There is a concentration of more recent studies, mainly between 2017 and 2023. This may be related to increased interest in the topic, especially after the COVID-19 pandemic, which intensified discussions about occupational health and absenteeism. However, even before the pandemic, presenteeism rates were already high in some contexts^27^.

The prevalence of presenteeism varied considerably, from 14.1% in Australia^29^ to 100% in Iran^39^, with many studies reporting rates above 50%. It is noteworthy that countries such as Australia (87.8%)^24^, Israel (72.3%)^38^, the United Kingdom^23^, and the United States^26^ (with variations between 68.6% and 92%) had significantly high prevalence rates of presenteeism, which may reflect greater institutional pressure to attend work even in poor health conditions, or indicate the presence of organizational cultures that are more permissive and tolerant of this practice.

As for the origin of presenteeism, the results suggest the influence of individual factors, such as professional commitment and fear of reprisals, and organizational factors, such as high workload and low staffing levels. These factors warrant careful interpretation, as the correlations between predictor variables and presenteeism can be ambiguous. For example, in one study, burnout and disengagement were correlated with a lower rate of presenteeism^11^, while in another, levels of burnout were correlated with higher presenteeism^15^.

Similarly, emotional stability has been associated with increased presenteeism^24^. This can be explained by the fact that workers who exhibit more stable mood behaviors tend to show up for work even when they are ill.

The findings indicate that higher presenteeism was also associated with an unfavorable organizational culture. In contrast, factors such as organizational commitment (β = −0.42, p < 0.001), support from coworkers (β = −0.15, p < 0.001)^28^, motivation in public service (β = −0.35; p < 0.001)^30^ and (β = −0.13, p < 0.01)^12^ and affective commitment (β = −0.27; *p* < 0.001) reduce the risk of presenteeism. In addition, 75% of professionals reported that the primary cause of this episode was organizational in nature^18^.

Workload also showed a strong association with presenteeism. Among the factors mentioned in the articles are excessive working hours (r=0.105, p=0.016)^36^, shift work (r=0.106, p=0.015)^36^, and workaholism (r=0.232, p<0.001)^36^.

During the pandemic, a review of the literature identified factors that influenced presenteeism, including issues directly related to mental health, individual factors arising from the COVID-19 pandemic, and working conditions^4^.

Demographic factors, such as age and gender, differed across the studies analyzed, preventing generalization. As these are individual factors, they deserve special attention in each analyzed context, given the cultural and socioeconomic differences across regions.

The most frequently cited reasons for presenteeism included mild symptoms (36/90; 40.0%) and difficulty in finding substitute health professionals to cover (25/90; 27.8%). Two nurses stated that their supervisors prohibited them from taking sick leave, even when they had symptoms. In total, 106 healthcare professionals came to work while sick, leading to exposure of up to 234 patients, of whom 137 subsequently received antiviral chemoprophylaxis^21^. The most common reason was a “sense of professional duty”^40^. These results align with a qualitative study conducted with Brazilian nurses, which found that professional commitment was a reason for presenteeism, in addition to financial need^41^.

As for the consequences for individual and organizational health, studies have confirmed links between presenteeism, unfavorable clinical outcomes, and poor quality of care, such as increased medical errors and compromised patient safety^18^ ^-^ ^20^ ^,^ ^27^ ^,^ ^31^ ^,^ ^34^. In one of these studies, the source of a respiratory infection outbreak in a small mental health treatment unit was attributed to the presenteeism of a single healthcare professional. The infections were spread by this professional, who went to work sick and infected a total of 17 patients. Of these, five developed complications from pneumonia, and a total of US$ 12,324 in additional medical costs was incurred because of the outbreak^31^.

These unfavorable results are the costs of presenteeism, which are often invisible to institutions, since, in most cases, assessments carried out do little to investigate in depth the variables underlying presenteeism and its consequences. It is necessary to understand this phenomenon from a systemic and multidimensional perspective, as the compromise of the health of the professional (both those who practice and those who work with them) reflects in the worsening of pre-existing diseases, fatigue, lower productivity, increased risk of future absenteeism, burnout, increased costs with future leave, chronic diseases, and intention to leave the job^18^ ^-^ ^19^. All these consequences, at the individual level, are reflected in consequences for patients and institutions, becoming a vicious cycle of organizational illness.

People who practice presenteeism may experience short-term health benefits, but their well-being may deteriorate over time^42^. Other studies report that presenteeism can have positive aspects and be functional in certain contexts when appropriate resources are available^43^. However, the findings of this research indicate that the consequences of presenteeism are predominantly negative for patient safety.

As for interventions to reduce presenteeism among hospital healthcare professionals, only two studies emphasized this topic^22^ ^-^ ^23^. These addressed interventions at the occupational health level and the creation of centers for professional well-being, but there were no more robust specific organizational interventions. This is noteworthy, given that environments with strong organizational support have been shown to promote greater self-esteem among workers and reduce presenteeism rates.

Wellness centers were facilities created during the COVID-19 pandemic to offer comfort through positive sensory elements (soft lighting, stress balls, essential oils) and access to psychological first aid for healthcare professionals fighting the coronavirus^22^ ^-^ ^23^.

Investments are needed in interventions that include commitment, mutual support, and organizational support as guidelines for hospital personnel management policies, which can involve professionals as protagonists in decision-making processes, workforce planning, and participation in discussions on issues related to presenteeism and its relationship to the quality of care.

In addition, interventions are suggested at both the organizational and individual levels. Among the organizational interventions, improving salary distribution, balancing workloads, and promoting rewards based on employee feedback are also noteworthy. It is essential to strengthen organizational support by creating an environment where workers feel valued, listened to, and supported, and by investing in sensitive, empathetic leaders committed to the team’s well-being. Promoting an organizational culture that values workers, along with implementing clear policies on sick leave and managing work overload, is also crucial.

At the individual level, it is recommended that selection processes include an assessment of emotional skills for work, which include self-awareness, self-control, self-actualization, and empathy. It is also important to offer ongoing training and psychological support to strengthen professionals’ self-esteem. Finally, internal awareness campaigns about the risks of presenteeism are essential to alert professionals to the negative impacts of working while sick, both for themselves and for their teams and patients.

Considering this, there is a clear need for advances in the formulation of institutional policies that prioritize organizational health and the promotion of positive practice environments, and that recognize presenteeism as a phenomenon that, if neglected, compromises both the care provided and the health of the professionals themselves. Hospital managers, therefore, play a fundamental role in reducing presenteeism. Conducting new studies, with a qualitative and longitudinal focus, is essential to deepen understanding of the causes and consequences of presenteeism and to support the development of effective interventions.

Among the limitations, some articles did not provide detailed information about the hospital sector or the participants’ professional category. In several cases, the studies refer generally to “health professionals” or “hospital workers,” without distinguishing between roles or areas of practice. This lack of detail limits the comparison between institutional contexts and specific categories within the hospital environment. Despite the severity of the impacts, few studies address interventions to prevent or mitigate presenteeism. The strategies identified were specific, such as the implementation of wellness centers, but there is no consensus on their effectiveness. In addition, the predominance of cross-sectional studies in this review also reveals limitations to the results.

## Conclusion

This scoping review fulfilled its mapping purpose and demonstrated that presenteeism is a prevalent and multifactorial phenomenon among healthcare professionals in hospital settings. The results indicate that professionals often attend work even when their health is compromised, motivated by factors such as organizational pressure, an institutional culture that values “working despite illness,” lack of support policies, fear of reprisals, and a sense of responsibility toward patients.

The consequences are far-reaching: on an individual level, presenteeism contributes to the worsening of chronic diseases, stress, fatigue, and reduced work capacity; at the organizational level, it compromises productivity, increases direct and indirect healthcare costs, and negatively impacts patient safety, with a higher risk of errors and the spread of infections.

## Data Availability

The dataset of this article is available on the RLAE page in the SciELO Data repository, at the link https://doi.org/10.17605/OSF.IO/GVNX3
