# Supplementary material for: Presenteeism among healthcare professionals in a hospital setting: a scoping review
Source: Rev Lat Am Enfermagem. 2026 Aug 10;34:e4900. doi: 10.1590/1518-8345.8286.4900 (PMC13459727; doi:10.1590/1518-8345.8286.4900)
Supplement: Translation

# Translation: Presenteísmo de profissionais da saúde em contexto hospitalar: uma revisão de escopo

**DOI:** 10.1590/1518-8345.8286.4901

**Authors:** Cynthia Lima Sampaio, Thailane Cardoso Soares, Victoria Lohreine de Carvalho Grangense, Bruna Victória da Silva Passos, Roberta Meneses Oliveira, Joselany Áfio Caetano

**Affiliations:** 1 Universidade Federal do Ceará, Departamento de Enfermagem, Fortaleza, CE, Brasil.

**Keywords:** Hospitais, Presenteísmo, Saúde Ocupacional, Gestão em Saúde, Pessoal de Saúde, Revisão de Escopo.

## Abstract

**Objetivo: mapear a prevalência do presenteísmo entre profissionais da saúde no ambiente hospitalar, identificar fatores relacionados, consequências para a segurança do paciente e a saúde do trabalhador, bem como as intervenções utilizadas.:**

**Método:**

revisão de escopo realizada com base nas recomendações do JBI *Reviewer’s Manual*, incluindo estudos disponíveis na íntegra em cinco bases de dados, sem restrição de tempo ou idioma.

**Resultados:**

os 31 artigos selecionados mostraram alta prevalência de presenteísmo entre profissionais da saúde no ambiente hospitalar e os principais fatores relacionados foram as doenças (depressão e infecções), aspectos ocupacionais (sobrecarga de trabalho) e organizacionais (falta de apoio dos colegas). As consequências para a saúde do trabalhador foram o agravamento da saúde, estresse, fadiga/exaustão, comprometimento da capacidade laboral, e da segurança do paciente, o aumento do risco de infecções e de erros clínicos. Poucos estudos relataram intervenções, como centros de bem-estar ocupacional. As consequências repercutiram nos aspectos econômicos com perda de produtividade e maior custo em saúde.

**Conclusão:**

a prevalência de presenteísmo entre profissionais da saúde em ambiente hospitalar é alta e constitui um fenômeno multifatorial com consequências negativas para indivíduos, organizações e pacientes. A análise multidimensional do presenteísmo pode contribuir para o estabelecimento de políticas e programas de saúde organizacional em hospitais. Frente à escassez de estudos de intervenção, é necessário avançar no conhecimento sobre o tema. O protocolo da pesquisa consta na plataforma Open Science Framework: https://doi.org/10.17605/OSF.IO/GVNX3.

## Destaques

**(1)** O presenteísmo é um fenômeno prevalente e multifatorial entre profissionais da saúde em ambiente hospitalar. **(2)** O presenteísmo contribui para o agravamento de doenças crônicas, estresse, fadiga e redução da capacidade laboral. **(3)** No âmbito organizacional, ele compromete a produtividade, eleva os custos diretos e indiretos com saúde. **(4)** Impacta negativamente o clima de segurança do paciente, com maior risco de erros e disseminação de infecções. **(5)** Estudos de intervenção são necessários avançar no conhecimento sobre o tema.

## Introdução

As instituições de saúde são reconhecidas como ambientes de trabalho de alta complexidade e exposição a riscos, abrangendo aqueles comuns a outros setores somados aos inerentes às práticas assistenciais. Os profissionais da saúde, especialmente aqueles que atuam em hospitais, estão expostos a uma ampla variedade de riscos, tais como biológicos, químicos, físicos, ergonômicos, além dos problemas organizacionais e riscos psicossociais^1^. 

Nesse contexto, o absenteísmo e o presenteísmo são resultados comuns frente à exposição ao risco e repercutem na produtividade e no bem-estar dos profissionais. No entanto, enquanto o absenteísmo entre profissionais de enfermagem é amplamente estudado devido ao seu elevado custo para os sistemas de saúde, o presenteísmo é um fenômeno mais complexo, de difícil mensuração, por envolver múltiplos fatores^2^.

É possível exemplificar os altos custos do absenteísmo com a perda anual de aproximadamente US$ 4 milhões para a Província Oriental da Arábia Saudita^2^. Já em relação ao presenteísmo, os estudos apresentam grande heterogeneidade metodológica e resultados contraditórios quanto às associações com variáveis ocupacionais ou organizacionais. Isso dificulta a formulação de conclusões definitivas no campo da saúde^3^.

Ademais, o presenteísmo ainda é um conceito em construção, mas já reconhecido como a presença do profissional no trabalho mesmo quando há limitações físicas e mentais que impeçam sua atividade laboral, embora também exista a classificação de presenteísmo não relacionado à doença devido a razões pessoais, como conflito entre vida profissional e pessoal, percepção de falta de apoio organizacional, estresse, entre outros^3^.

O cenário pós-pandemia de COVID-19 ampliou o interesse por esse fenômeno, uma vez que os trabalhadores de saúde enfrentaram condições excepcionais de carga emocional, física e organizacional, com possíveis repercussões duradouras sobre o presenteísmo, a segurança do paciente e a saúde ocupacional^4^. Estudos recentes apontam taxas de prevalência elevada de presenteísmo entre profissionais em ambiente hospitalar, relacionadas à exaustão e repercussão na qualidade do cuidado e na segurança do paciente^4^ ^-^ ^5^.

A despeito das evidências práticas sobre a repercussão do presenteísmo nos serviços de saúde, faltam estudos que articulem, de forma mais aprofundada, a prevalência, os fatores relacionados e as consequências e intervenções específicas no contexto hospitalar.

O presente estudo justifica-se diante da necessidade de mapear a relação entre presenteísmo, esgotamento físico e mental, exaustão, síndrome de *burnout*, estresse e depressão, para permitir intervenções eficazes. 

No campo da Enfermagem, a relevância deste estudo consiste no fato dos trabalhadores de enfermagem representarem a maior força de trabalho nas instituições de saúde e desempenharem papel central na assistência direta ao paciente. O presenteísmo entre profissionais de enfermagem pode acarretar implicações para a segurança do cuidado ofertado, a qualidade da assistência de enfermagem e aumento dos custos organizacionais.

Compreender o presenteísmo sob a perspectiva da gestão em enfermagem é essencial para subsidiar intervenções que promovam ambientes de trabalho mais saudáveis, com redução dos riscos ocupacionais e que favoreçam práticas de cuidado seguro.

Portanto, objetivou-se mapear a prevalência do presenteísmo entre profissionais da saúde no ambiente hospitalar, identificar fatores relacionados, consequências para a segurança do paciente e a saúde do trabalhador, bem como as intervenções utilizadas.

## Método

### Tipo de estudo

O estudo trata de uma revisão de escopo que seguiu as etapas recomendadas pelo JBI *Reviewer’s Manual*, desenvolvida mediante cinco etapas: formulação da questão de pesquisa; identificação dos estudos relevantes; seleção de estudos; extração e análise dos dados; síntese e construção do relatório. Utilizou-se o *Preferred Reporting Items for Systematic Reviews* and *Meta-Analyses Extension for Scoping Reviews* (PRISMA-ScR) para a redação do estudo. O registro do protocolo da pesquisa consta na plataforma *Open Science Framework* (OSF) sob o *Digital Object Identifier* (DOI) https://doi.org/10.17605/OSF.IO/GVNX3. A busca nas diferentes bases de dados foi realizada em setembro de 2024. 

### Questão de pesquisa

A pergunta de estudo foi elaborada por meio do uso do acrônimo PCC, segundo as recomendações do JBI. Considerou-se, como população (P), profissionais da saúde; conceito de interesse (C), presenteísmo; e, como contexto (C), hospital; formulando-se a seguinte questão: “Qual é o panorama da temática presenteísmo de profissionais da saúde no ambiente hospitalar? ”

Para responder à questão norteadora, outros questionamentos foram realizados: P1. Qual é a prevalência do presenteísmo entre profissionais da saúde no ambiente hospitalar? P2. Quais são os fatores relacionados ao presenteísmo nessa população?; P3. Quais são as consequências do presenteísmo para a segurança do paciente e para a saúde do profissional no ambiente hospitalar?; e P4. Quais intervenções individuais e organizacionais são sugeridas para a redução do presenteísmo no ambiente hospitalar?

### Critérios de seleção

A revisão abordou estudos publicados na íntegra que respondessem à questão de pesquisa. Os critérios de inclusão foram: publicações sem restrição de tempo ou de idioma, com texto completo disponível. Excluíram-se editoriais, vídeos, sites, notícias, *preprints* e resumos. 

### Estratégia de busca

A busca foi realizada nas bases de dados: MEDLINE/PubMed (via *National Library of Medicine*), *Web of Science*, CINAHL (via EBSCO), *Scopus* e *Embase*, nas seguintes etapas: 1) pesquisa inicial no MEDLINE para identificar estudos sobre o assunto e selecionar as palavras contidas nessas publicações; 2) elaboração da estratégia de busca; 3) validação da estratégia de busca elaborada com auxílio de uma bibliotecária; 4) uso da nova estratégia. 

A estratégia de busca adotada inicialmente foi “presenteeism” OR “sickness presence” AND “hospitals” AND “healthcare workers”. Após consulta com a bibliotecária, a estratégia ficou definida da seguinte forma, utilizando-se o MeSH *(Medical Subject Headings)*: ((Presenteeism OR “Sickness Presence” OR “Presence, Sickness”) AND (Hospitals OR “Hospital Administration”) AND (“Health Personnel” OR “Personnel, Health” OR “Healthcare Workers” OR “Healthcare Worker” OR “Health Care Providers” OR “Health Care Provider” OR “Provider, Health Care” OR “Healthcare Providers” OR “Healthcare Provider” OR “Provider, Healthcare” OR “Health Care Professionals” OR “Health Care Professional” OR “Professional, Health Care”)) e da seguinte maneira, utilizando-se o EMTREE: ((‘presenteism’ OR ‘sickness presence’ OR ‘working while ill’ OR ‘working while sick’ OR ‘presenteeism’) AND (‘health care practitioner’ OR ‘health care professional’ OR ‘health care provider’ OR ‘health care worker’ OR ‘health personnel’ OR ‘health profession personnel’ OR ‘health worker’ OR ‘healthcare personnel’ OR ‘healthcare practitioner’ OR ‘healthcare professional’ OR ‘healthcare provider’ OR ‘healthcare worker’ OR ‘home health aides’ OR ‘personnel, health’ OR ‘public health officer’ OR ‘health care personnel’) AND hospital).

A estratégia com descritores cadastrados no EMTREE foi utilizada na base de dados Embase e a estratégia com descritores cadastrados no MeSH foi utilizada nas demais bases selecionadas. O acesso às bases de dados deu-se por meio do Portal de Periódicos da Coordenação de Aperfeiçoamento de Pessoal de Nível Superior (CAPES).

Cabe ressaltar que foi realizada pesquisa nas plataformas *International Prospective Register of Systematic Reviews* (PROSPERO) e OSF para conferir a existência de revisões sobre o objeto de estudo, porém não foram identificadas. 

Uma nova busca na Biblioteca Digital Brasileira de Teses e Dissertações (BDTD) e nos Catálogos de teses e dissertações da CAPES foi realizada em novembro de 2025, utilizando a seguinte estratégia: *Presenteeism* AND *Hospitals* AND “*Health Personnel*”, sendo encontrado apenas um estudo, repetido nos dois repositórios. Esse estudo já havia sido contemplado na busca inicial nas bases de dados. 

### Seleção dos estudos

Após a pesquisa nas bases de dados, todos os registros identificados foram agrupados e carregados no aplicativo Rayyan (*Intelligent Systematic Review*)^6^. E, no primeiro passo, as duplicatas foram removidas. Após essa retirada, os títulos e os resumos foram lidos e selecionados por dois revisores independentes para avaliação, aplicando os critérios de inclusão e exclusão. Os estudos potencialmente elegíveis foram obtidos em texto completo e avaliados pelos mesmos revisores. As discordâncias quanto aos estudos selecionados foram discutidas entre os revisores e, quando não houve consenso, um terceiro revisor foi acionado. 

Os revisores participantes possuem formação em Enfermagem e integram um Núcleo de Ensino, Pesquisa e Extensão em Gestão e Cuidado em Saúde de uma universidade federal, na linha de pesquisa: Atenção e Gestão do cuidado em saúde organizacional e segurança do paciente. A etapa de triagem e seleção foi conduzida, inicialmente, por dois revisores independentes, sendo um estudante de doutorado com experiência em revisão de escopo e um membro de iniciação científica. O primeiro revisor selecionou 31 estudos e o segundo, 15, apresentando concordância em 12 registros, o que corresponde a 35% de concordância. Diante da divergência observada, um terceiro revisor, estudante de mestrado, com experiência em revisão de escopo, realizou nova triagem e selecionou 29 estudos, todos coincidentes com as escolhas do primeiro revisor, resultando em concordância total entre ambos (kappa = 1,00). Os motivos de exclusão em texto completo foram registrados e apresentados no fluxograma PRISMA-ScR. 

### Instrumento utilizado para a coleta das informações

A extração dos dados foi realizada com instrumento desenvolvido pelas autoras, baseado no formulário sugerido pelo manual JBI e em publicações de protocolo, sendo adaptado para o objeto de estudo^7^. Os dados extraídos foram: identificação do estudo (autor, título, ano, país, desenho/delineamento, objetivo); participantes (número total, formação profissional, local do estudo); resultados encontrados (prevalência do presenteísmo de trabalhadores da saúde no ambiente hospitalar, fatores relacionados, consequências para a segurança do paciente e a saúde do trabalhador, intervenções utilizadas para diminuir o presenteísmo); conclusões. 

As informações foram organizadas em planilha eletrônica no Microsoft Excel^®^, permitindo o controle das etapas e a verificação cruzada dos dados. Nenhum dado foi alterado ou excluído sem acordo entre os revisores.

### Tratamento e análise dos dados

Com base nos dados extraídos, realizou-se a análise descritiva e foram construídos quadros e figuras com os dados das publicações. Os resultados da pesquisa e do processo de inclusão do estudo encontram-se relatados, na íntegra, na revisão de escopo final e apresentados em um fluxograma *Preferred Reporting Items for Systematic Reviews and Meta-analyses extension for scoping review* (PRISMA-ScR). 

A síntese dos resultados foi organizada de forma narrativa, agrupando os achados conforme semelhanças conceituais e metodológicas. Optou-se por não realizar avaliação crítica da qualidade metodológica, em consonância com as recomendações do JBI para revisões de escopo. 

Os fatores que influenciam o presenteísmo foram identificados nos estudos por meio de testes estatísticos de associação e de correlação, incluindo: qui-quadrado (χ²), t de Student e ANOVA para associações e correlação de Pearson (r) e correlação de Spearman para medir correlações^8^.

### Aspectos éticos

O presente estudo não envolveu seres humanos e os dados utilizados são considerados secundários de domínio público com livre acesso. Além disso, foram inseridas todas as referências com respeito aos direitos autorais^9^. Portanto, não houve a necessidade de submissão ao Comitê de Ética e Pesquisa.

## Resultados

As buscas resultaram em 216 estudos. Após a exclusão dos duplicados, restaram 123 estudos para a seleção. Na análise dos títulos e resumos, foram selecionados 56; e, após a leitura integral dos estudos, 31 foram incluídos na revisão (Figura 1).

**Figura 1:** Fluxograma PRISMA utilizado para identificação e seleção dos estudos. Fortaleza, CE, Brasil, 2025

Fonte: Fluxograma PRISMA de seleção de estudos

Os 31 estudos apresentam ampla diversidade quanto ao país de origem, ano de publicação, delineamento metodológico e instrumentos utilizados (Figura 2). A maioria é composta por estudos transversais (n = 22; 70,97%), seguida de três estudos longitudinais prospectivos (9,68%) e três retrospectivos (9,68%). Além disso, identificou-se um estudo metodológico (3,23%), uma revisão sistemática (3,23%) e uma carta ao editor (3,23%).

**Figura 2:** Identificação dos estudos incluídos segundo código, país, ano de publicação, delineamento metodológico, perguntas respondidas na revisão e instrumentos utilizados. Fortaleza, CE, Brasil, 2025

Código do artigo	País/Ano	Desenho	Resposta às Questões de Revisão (P1 a P4)	Instrumentos de medida
A1^10^	Suíça, 2021	Longitudinal prospectivo	P1	*Charlson Comorbidity Index* Histórico de vacinação contra gripe e características domiciliares que poderiam interferir no risco de gripe Diários de sintomas respiratórios e adesão ao uso de máscara
A2^11^	Nigéria, 2021	Transversal	P1 e P2	*Oldenburg Burnout Inventory* (OLBI) *Stanford Presenteeism Scale* (SPS-6) *Patient Health Questionnaire-2* (PHQ-2)
A3^12^	China, 2021	Transversal	P2	*Perceived Ability to Work Scale* (PAWS) *Challenge and Hindrance-related Self-Reported Stress Scale* (C-HSS) *Short Form Health Survey* (SF-8) *Public Service Motivation Scale*
A4^13^	China, 2019	Transversal	P1 e P2	*Perceived Ability to Work Scale* (PAWS) *Short Form Health Survey* (SF-8) *Challenge and Hindrance-related Self-Reported Stress Scale* (C-HSS) *Short-form PSM Scale* *Job in General Scale* *Turnover Intention Scale* *Job Performance Scale* *Chirurgisches Qualitätssiegel Scale*
A5^14^	China, 2022	Transversal	P1 e P2	Escala de Niehoff & Moorman (justiça distributiva); Escala de Pierce, et al (autoestima organizacional) *Perceived Ability to Work Scale* (PAWS) Escala de Eisenberger, et al. (Suporte Organizacional Percebido)
A6^15^	Turquia, 2024	Metodológico	P2	*Stanford Presenteeism Scale* (STAS) *Happiness At Work Scale* *Burnout Scale Short Version*
A7^16^	China, 2024	Transversal	P2	*Impact of Events Scale* (IES) *Turnover intention Scale* (PTSD) *Stanford presenteeism Scale* (STAS) *Social Support Scale* (SSS)
A8^17^	Brasil, 2021	Transversal	P1 e P2	*Stanford Presenteeism Scale* (STAS) - versão brasileira *Safety Attitudes Questionnaire* (SAQ)
A9^18^	Malta, 2020	Transversal	P2 e P3	Questionário sobre as percepções de doenças durante episódios de presenteísmo e absenteísmo *Brief Illness Perception Questionnaire* (B-IPQ)
A10^19^	França, 2022	Revisão sistemática	P3	*Stanford Presenteeism Scale* (SPS-6) *Work Limitations Questionnaire* (WLQ) *Work Productivity and Activity Impairment* (WPAI) *Health and Work Performance Questionnaire* (HPQ) *Perceived Ability to Work Scale* (PAWS)
A11^20^	Brasil, 2022	Transversal	P1, P2 e P3	*Stanford Presenteeism Scale* (SPS-6) *Work Ability Index* (WAI)
A12^21^	Japão, 2020	Carta ao editor	P1	Duas perguntas abertas sobre sintomas, reconhecimento da doença e motivos para comparecer ao trabalho doente.
A13^22^	Reino Unido, 2024	Transversal	P4	*Warwick-Edinburgh Mental Wellbeing Scale* (WEMWBS) Quatro medidas globais de item único de estresse no trabalho, satisfação no trabalho, presenteísmo e intenções de rotatividade.
A14^23^	Reino Unido, 2020	Transversal	P1 e P4	*Warwick-Edinburgh Mental Wellbeing Scale* (WEMWBS) Escalas globais de item único (estresse, satisfação, intenção de saída e presenteísmo); *Utrecht Work Engagement Scale* (UWES - subescala dedicação, 3 itens) Questões abertas sobre percepção, benefícios e barreiras dos centros
A15^24^	Austrália, 2021	Transversal	P1 e P2	Pergunta sobre quantas vezes compareceram ao trabalho quando estavam doentes ou não se sentindo bem nos últimos 12 meses.
A16^25^	China, 2017	Transversal	P1 e P2	*Perceived Ability to Work Scale* (PAWS)
A17^26^	Estados Unidos, 2017	Transversal	P1	Questionário autorrelatado online e anônimo
A18^27^	Portugal, 2016	Longitudinal retrospectivo	P1 e P3	*Work Limitations Questionnaire8* (WLQ8) (versão abreviada de 8 itens) *Stanford Presenteeism Scale* (SPS6)* *
A19^28^	China, 2019	Transversal	P1 e P2	*Perceived Ability to Work Scale* (PAWS) *Short Form Health Survey* (SF-8) *Challenge and Hindrance-related Self-Reported Stress Scale* (C-HSS) *Short-form PSM scale* Escala de Ironson, et al. (satisfação no trabalho) Escala de Singh, et al. (intenção de rotatividade) Escala de Darwish (desempenho no trabalho) *Chirurgisches Qualitätssiegel Scale*
A20^29^	Austrália, 2020	Longitudinal retrospectivo	P1	Presenteísmo inferido por ausência de registro de licença médica durante o período de infecção confirmada Absenteísmo medido por horas de licença registradas / horas totais agendadas × 100 Dados laboratoriais, administrativos e de folha de pagamento integrados via número de identificação do funcionário.
A21^30^	China, 2019	Transversal	P2	*Perceived Ability to Work Scale* (PAWS)
A22^31^	Japão, 2022	Longitudinal retrospectivo	P3	Não foi usado nenhum questionário ou escala padronizada de presenteísmo
A23^32^	Croácia, 2017	Transversal	P1 e P2	*Patient Safety Culture;* *Health and Work Performance Questionnaire* (HPQ)* *
A24^33^	China, 2018	Transversal	P1 e P2	*Perceived Ability to Work Scale* (PAWS) *Challenge and HindranceRelated SelfReported Stress Scale*
A25^34^	Eslovênia, 2020	Transversal	P1 e P3	Questionário desenvolvido pelos próprios autores, com várias perguntas sobre saúde, trabalho e comportamento
A26^35^	Espanha, 2018	Transversal	P1 e P2	*Stanford Presenteeism Scale6* (SPS-6)* *
A27^36^	Japão, 2020	Transversal	P1 e P2	Questionário elaborado pelo autor para variáveis demográficas e sintomas *Work Productivity and Activity Impairment - General Health* (WPAIGH)
A28^37^	Tailândia, 2022	Longitudinal prospectivo	P1	Registro direto por observadores durante o turno e questionário com autorrelato de sintomas nos últimos sete dias
A29^38^	Israel, 2024	Longitudinal prospectivo	P1	Relatos dos participantes, combinados com os dados de vigilância ativa
A30^39^	Irã, 2022	Transversal	P1 e P2	*Valuation of Lost Productivity Questionnaire* (VOLP), adaptado para COVID-19 *WHO STEPwise*
A31^40^	Estados Unidos, 2019	Transversal	P1 e P2	Questionário anônimo desenvolvido pelos autores com itens fechados sobre razões do presenteísmo

Quanto aos países, houve predominância de estudos conduzidos na China (n = 8; 25,81%), seguidos por Japão (n =3; 9,68%), Austrália (n = 2; 6,46%), Brasil (n = 2; 6,45%), Reino Unido (n = 2; 6,45%), Estados Unidos (n = 2; 6,45%) e 1 (3,23%) estudo na Suíça, Nigéria, Malta, Turquia, França, Portugal, Croácia, Eslovênia, Espanha, Tailândia, Israel e Irã. Em relação ao ano de publicação, observou-se maior concentração em 2020 (n = 6; 19,35%) e 2022 (n = 6; 19,35%), seguidos de 2021 (n = 5; 16,13%), 2019 (n = 4; 12,90%) e 2024 (n = 4; 12,90%). Os demais anos apresentaram menor frequência: 2017 (n = 3; 9,68%), 2018 (n = 2; 6,45%) e 2016 (n = 1; 3,23%). 

Os instrumentos utilizados para avaliar o presenteísmo incluíram escalas padronizadas, como a *Perceived Ability to Work Scale* (PAWS) (n= 8), *Stanford Presenteeism Scale* (SPS-6) (n= 7), *Work Limitations Questionnaire* (WLQ) (n= 2), *Work Productivity and Activity Impair*ment (WPAI) (n= 2), *Health and Work Performance Questionnaire* (HPQ) (n= 2) e *Valuation of Lost Productivity Questionnaire* (VOLP), adaptado para COVID-19 (n=1). Além dessas, alguns estudos aplicaram questionários próprios contendo perguntas diretas sobre a frequência do comportamento de presenteísmo e outros instrumentos de medida de constructos associados, como *Burnout*, Motivação para o serviço, Felicidade no trabalho, Suporte Social, Estresse de Mudança, *Turnover*, dentre outros (n= 26).

Dos 31 artigos selecionados, 23 responderam à pergunta sobre o resultado da medição de presenteísmo (P1), conforme a Figura 3. Os estudos analisam o presenteísmo sob diferentes perspectivas, como trabalhar doente ou perda de produtividade, utilizando escalas variadas. Alguns estudos fornecem dados sobre a prevalência do presenteísmo e a intensidade com que ele ocorre. Já outros divulgam apenas a prevalência, a média ou a unidade de medida (perda de produtividade, grau ou valor financeiro).

**Figura 3:** Estudos sobre a prevalência do presenteísmo entre profissionais do contexto hospitalar, por país, ano, número de participantes, instrumentos utilizados e principais resultados. Fortaleza, CE, Brasil, 2025

Stanford Presenteeism Scale (SPS-6)			
País, ano	Número de participantes	Resultados sobre presenteísmo	
		Prevalência	Média
Nigéria, 2021^11^	155	não informada	22,1 (DP* 5,1)
Espanha, 2018^35^	323	52,9%	19,84 (DP* 4,2)
Brasil, 2021^17^	754	43,5%	20,9 (DP* 4,7)
Brasil, 2022^20^ ^)^	299	65,6%	21,36 (DP* 4,7)
**Perceived Ability to Work Scale (PAWS)**			
China, 2017^25^	1392	não informada	7,25 (DP* 1,79) a 7,63 (DP* 1,57)
China, 2019^13^	572	não informada	3,92 (DP* 2,33) a 4,12 (DP* 2,38)
China, 2019^28^	1434	não informada	7,44 (DP* 1,716) a 7,71 (DP* 1,59)
China, 2018^33^	1392	não informada	2,37 (DP* 1,67) a 2,75 (DP* 1,79)
China, 2022^14^	1122	não informada	2,09 (DP* 1,46) a 2,35 (DP* 1,61)
**Work Limitations Questionnaire- 8 (WLQ-8) e SPS-6**			
Portugal, 2016^27^	188	não informada	*WLQ-8*: Perda de 19,56% de produtividade por dia útil. *SPS-6*: enfermeiros 1,95 (0,96) e auxiliares 2,28 (0,70)
**Work Productivity and Activity Impairment-General Health (WPAI-GH)**			
Japão, 2020^36^	668	62,1%	O grau de presenteísmo foi de 30
**Valuation of Lost Productivity Questionnaire (VOLP) adaptado para COVID-19**			
Irã, 2022^39^	250	100%	US$ 1.489,05 (DP* = US$ 1.143,15) no período de três meses.
**Health and Work Performance Questionnaire (WHO-HPQ)**			
Croácia, 2017^32^	595	70%	6,82%
**Questões elaboradas pelo autor sobre percepção do presenteísmo**			
Japão, 2020^21^	90	Prevalência de 48,9%	
Reino Unido, 2020^23^	819	Prevalência de 68,6%	
Austrália, 2021^24^	320	Prevalência de 87,8%	
Estados Unidos, 2017^26^	286	Prevalência de 92,0%	
Israel, 2024^38^	2505	Prevalência de 72,3% (IC^†^ de 95%: 60,4-86,4).	
Eslovênia, 2020^34^	5865	Prevalência de 57,0%	
Tailândia, 2022^37^	240	Prevalência de 23,0%	
**Análise dos casos de gripe confirmados em laboratório**			
Austrália, 2020^29^	1.540	Prevalência de 14,1%	
Suíça, 2021^10^	152	Prevalência de 67,9%	
**Outras escalas**			
Japão, 2020^40^	14	Prevalência de 64,0%	

*DP = Desvio-padrão; ^†^IC = Intervalo de confiança

Os resultados mostram ampla variação na prevalência do presenteísmo, com oscilação de 14,1% na Austrália a 100% no Irã. Em relação às médias das escalas, observou-se variações de 2,37 (DP = 1,67) a 7,71 (DP = 1,59) na *PAWS*, e de 19,84 (DP = 4,2) a 22,1 (DP = 5,1) na *SPS-6*. 

Apenas três estudos compararam o presenteísmo entre diferentes categorias profissionais. Em um deles, os enfermeiros apresentaram menor nível de presenteísmo (14,48%) em comparação com os técnicos e auxiliares de enfermagem assistentes operacionais (27,19%)^27^. Em outro, o presenteísmo foi mais elevado entre os médicos^39^. Já uma pesquisa brasileira encontrou menor perda de produtividade entre médicos, quando comparados com todos os profissionais (23,85 pontos), seguidos dos técnicos de enfermagem e enfermeiros, que obtiveram escores de 20,91 pontos e 20,65 pontos, respectivamente, indicando uma menor redução em sua produtividade diante de problemas de saúde^20^.

Dezenove artigos responderam sobre os fatores que podem influenciar o presenteísmo (P2). Esses dados foram encontrados nos artigos por meio de associações e correlações (Figura 4). Um estudo com questão direta sobre o motivo de trabalhar doente teve maior porcentagem (56%) de resposta para “senso de dever como profissional de saúde”^40^.

**Figura 4:** Fatores que podem influenciar o presenteísmo verificados por meio de associações e correlações nos estudos revisados. Fortaleza, CE, Brasil, 2025

Associações significativas encontradas entre presenteísmo e fatores identificados nos estudos revisados
**Fatores demográficos** 🚬Tabagismo (Fumantes com maior presenteísmo: OR* = 2,99, p = 0,030)^(39)^ 👴Idade (Meia-idade: OR* = 11.189, p = 0,046)^(39)^ 👴Idade (30 anos ou menos, p = 0,023)^(15)^ 💍Solteiro (p = 0,014)^(15)^ 🏘Morar sozinho (p = 0,030)^(15)^ 🚻Sexo (Mulheres com risco 1,88 vezes maior, IC^†^: 1,06-3,32, p = 0,03)^(20)^ 🚻Sexo (Homens) (p < 0,05)^(13)^
**Fatores de saúde-doença** 🤯Estresse (p < 0,01)^(35)^ 😔Depressão (p < 0,015)^(11)^ 💪Doenças musculoesqueléticas (p < 0,05)^(18)^ ❤Doenças crônicas (p < 0,05)^(18)^ 🤕Dores de cabeça/enxaquecas (p < 0,05)^(18)^ 🦠COVID-19 (uma infecção: OR* = 6.816, p = 0,000), duas infecções: OR* = 24.023, p = 0,001)^(39)^
**Fatores ocupacionais** 🏥Urgência hospitalar (p = 0,004)^(35)^ 📊Clima de segurança negativo (p < 0,05)^(17)^ 💊Cultura de segurança do paciente^(32)^ 📈Carga e tempo de carreira (Profissionais em meio/fim de carreira)^(13)^ 📈Trabalhar 9 horas ou mais diariamente (p < 0,001)^(15)^ 📈5 anos ou menos na profissão (p = 0,012)^(15)^ 💰 Renda (Terceiro e quarto quartis com maior presenteísmo)^(39)^ 🩺Médico (p = 0,005)^(15)^ ⚖Justiça na distribuição de recompensas e reconhecimento (ex.: salários, carga de trabalho, promoções) têm níveis mais altos de presenteísmo^(14)^
**Correlações significativas encontradas entre presenteísmo e fatores identificados nos estudos revisados**
**Carga de trabalho** Exaustão (-0,350, p < 0,001)^‡^ ^(11)^ Desengajamento (-0,475, p < 0,001)^‡^ ^(11)^ Horas de trabalho excessivas (r = 0,105, p = 0,016)^§^ ^(36)^ Trabalho em turnos (r = 0,106, p = 0,015)^§^ ^(36)^ *Workaholism* (r = 0,232, p < 0,001)^§^ ^(36)^
**Comprometimento organizacional** Comprometimento organizacional (β = − 0,42, p < 0,001)^‡^ ^(28)^ Apoio dos colegas de trabalho (β = − 0,15, p < 0,001)^‡^ ^(28)^ Motivação no serviço público (β = − 0,35; p < 0,001)^‡^ ^(30)^ e (β = −0,13, p < 0,01)^‡^ ^(12)^ Comprometimento afetivo (β = −0,27; p < 0,001)^‡^ ^(25)^ Felicidade no trabalho (r = −0,567, p < 0,001)^‡^ ^(15)^
**Saúde-doença** Estresse de desafio (β = 0,07, p < 0,01)^§^ ^(12)^ Estresse de desafio (-0,05, p = 0,001)^‡^ ^(33)^ Estresse de impedimento (β = 0,13, p < 0,01)^§^ ^(12)^ e (0,25, p = 0,001)^§^ ^(33)^ Saúde (β = −0,12, p < 0,01)^‡^ ^(12)^ e (r = -0,35)^‡^ ^(33)^ Estresse de desafio e estresse de impedimento (r = 0,20, r = 0,32)^§^ ^(25)^ TEPT^||^ (β = 0,638, p < 0,001)^§^ ^(16)^ Intensidade da dor lombar (r = 0,248, p < 0,001)^§^ ^(36)^, Duração do sono (-0,131, p = 0,003)^‡^ ^(36)^ Sintomas depressivos (r = 0,269, p < 0,001)^§^ ^(36)^ Estabilidade emocional (r = -0,226, p ≤ 0,01)^‡^ ^(24)^ Estresse relacionado ao trabalho (r= 0,218, p ≤ 0,001)^§^ ^(24)^ *Burnout* (r= 0,653, p < 0,001)^§^ ^(15)^

*OR = *Odds Ratio*; ^†^IC = Intervalo de confiança; ^‡^Correlação negativa; ^§^Correlação positiva; ^||^TEPT = Transtorno do estresse pós-traumático

Os fatores de associação identificados foram subdivididos em: demográficos, saúde-doença e ocupacionais, revelando a importância de idade, sexo, tabagismo, doenças físicas e mentais, renda, setor, carga horária e clima de segurança. 

Identificou-se que a presença de fatores como exaustão, desengajamento, comprometimento organizacional, apoio dos colegas de trabalho, motivação no serviço público, comprometimento afetivo, felicidade no trabalho, melhor saúde geral, maior duração do sono e estabilidade emocional foram correlacionados a menor presenteísmo.

Por outro lado, maior carga horária de trabalho, trabalho em turnos, estresse de desafio e de impedimento, sintomas depressivos, maior intensidade de dor lombar e presença de sintomas de TEPT (Transtorno do estresse pós-traumático) foram correlacionados a maior presenteísmo.

A respeito das consequências do presenteísmo (P3), encontraram-se sete artigos que responderam sobre as repercussões para a segurança do paciente e do trabalhador e também sobre o impacto na capacidade laboral e econômica, conforme representado na Figura 5.

**Figura 5:** Mapa mental sobre as consequências do presenteísmo. Fortaleza, CE, Brasil, 2025

No que se refere às intervenções individuais e organizacionais adotadas (P4), apenas dois estudos relataram a utilização de centros de bem-estar como estratégia institucional. Em uma dessas investigações, observou-se que profissionais de saúde sem acesso a centros de bem-estar apresentaram níveis mais elevados de presenteísmo (*β* = −0,22; *p* < 0,001) e eram, em média, mais jovens (*β* = 0,09; *p* = 0,002). Além disso, foi identificada uma interação significativa entre presenteísmo e acesso ao centro de bem-estar nos níveis de bem-estar dos profissionais, com *F*(1, 791) = 18,65; *p* < 0,001; ηp² = 0,02. Os profissionais que apresentaram presenteísmo e acessaram o centro relataram maiores níveis de bem-estar (média = 3,30; desvio-padrão - DP = 0,04), quando comparados àqueles que acessaram o centro, mas não relataram presenteísmo (média = 3,06; DP = 0,040)^22^.

Por outro lado, um segundo estudo não identificou diferenças estatisticamente significativas entre os profissionais que acessaram ou não os centros de bem-estar quanto às variáveis: percepção de estresse no trabalho, satisfação profissional, presenteísmo ou intenção de rotatividade^23^.

## Discussão

Os resultados apresentados permitem visualizar um panorama abrangente sobre diversos aspectos do presenteísmo. As perguntas norteadoras sobre a prevalência do presenteísmo e os fatores relacionados foram amplamente respondidas por meio do mapeamento realizado. No entanto, as respostas às perguntas sobre consequências e intervenções foram encontradas em poucos estudos.

Os achados reforçam a heterogeneidade de instrumentos para mensuração do presenteísmo e a importância de considerar múltiplos fatores contextuais e metodológicos ao interpretar os resultados. Em relação à prevalência de presenteísmo avaliada, os resultados variam conforme o instrumento de medida utilizado.

A PAWS foi a escala mais utilizada para investigações sobre a perda de produtividade, seguida da SPS-6. O instrumento original considera a autopercepção sobre a capacidade de trabalho, com resultados que variam de 0 a 10, em que valores mais altos indicam maior capacidade de trabalho e melhor desempenho. Nos estudos incluídos nesta revisão, os autores invertem os escores para uma interpretação mais intuitiva, em que escores mais altos representam maior presenteísmo, enquanto escores mais baixos refletem menor presenteísmo ou maior capacidade percebida para o trabalho. Já a SPS-6 permite mensurar a influência de problemas de saúde na qualidade de trabalho e no desempenho do profissional, mostrando-se um instrumento de fácil entendimento, o que favorece seu preenchimento e análise^15^. 

Há uma concentração de estudos mais recentes, principalmente entre 2017 e 2023. Isso pode estar relacionado ao aumento do interesse no tema, especialmente após a pandemia de COVID-19, que intensificou as discussões sobre saúde ocupacional e absenteísmo. Contudo, mesmo antes da pandemia, as taxas de presenteísmo já eram elevadas em alguns contextos^27^.

A prevalência de presenteísmo variou consideravelmente, de 14,1% na Austrália^29^, a 100%, no Irã^39^, com muitos estudos mostrando taxas superiores a 50%. Destaca-se que países como Austrália (87,8%)^24^, Israel (72,3%)^38^, Reino Unido^23^ ^)^ e Estados Unidos^26^ (com variações entre 68,6% e 92%) apresentaram prevalências significativamente elevadas de presenteísmo, o que pode refletir uma maior pressão institucional para o comparecimento ao trabalho mesmo em condições de saúde inadequadas, ou ainda indicar a presença de culturas organizacionais mais permissivas e tolerantes a essa prática.

Quanto à origem do presenteísmo, os resultados encontrados sugerem a influência de fatores individuais, como compromisso profissional e medo de represálias, e de fatores organizacionais, como alta carga de trabalho e escassez de recursos humanos baixos níveis de pessoal. Esses fatores merecem interpretação cuidadosa, visto que as correlações encontradas entre variáveis preditoras e o desfecho presenteísmo podem ser ambivalentes. Por exemplo, para um estudo, o *burnout* e o desengajamento foram correlacionados a um menor índice de presenteísmo^11^, enquanto para outro, os níveis de *burnout* estiveram correlacionados a maior presenteísmo^15^. 

Da mesma forma, a estabilidade emocional foi associada a um aumento do presenteísmo^24^. Isso pode ser explicado pelo fato de os trabalhadores que apresentam comportamentos mais estáveis de humor tenderem a comparecer ao trabalho mesmo quando estão doentes.

Os achados indicam que o maior presenteísmo também esteve associado a uma cultura organizacional desfavorável. Em contrapartida, fatores como comprometimento organizacional (β = − 0,42, p < 0,001), apoio dos colegas de trabalho (β = − 0,15, p < 0,001)^28^, motivação no serviço público (β = − 0,35; p < 0,001)^30^ e (β = −0,13, p < 0,01)^12^ e comprometimento afetivo (β = −0,27; *p* < 0,001) reduzem o risco de presenteísmo. Ademais, 75% dos profissionais relataram que a causa primária desse episódio foi de natureza organizacional^18^. 

A carga de trabalho também mostrou forte associação com o presenteísmo. Dentre os fatores mencionados nos artigos, encontram-se as horas de trabalho excessivas (r=0,105, p=0,016)^36^, o trabalho em turnos (r=0,106, p=0,015)^36^, e o *workaholism* (r=0,232, p<0,001)^36^.

Durante a pandemia, uma revisão da literatura identificou fatores que influenciaram o presenteísmo, incluindo questões diretamente relacionadas à saúde mental, fatores individuais, fatores relacionados com a situação causada pela pandemia de COVID-19 e condições de trabalho^4^.

Os fatores demográficos, como idade e sexo, foram divergentes nos estudos analisados, não permitindo generalização. Por se tratar de fatores de natureza individual, merecem atenção especial em cada contexto analisado, considerando as diferenças culturais e socioeconômicas de cada região.

As razões mais frequentemente citadas para o presenteísmo incluíram sintomas leves (36/90; 40,0%) e dificuldade em encontrar profissionais de saúde para substituí-los (25/90; 27,8%). Dois enfermeiros declararam que seus supervisores os proibiram de tirar licença médica, mesmo apresentando sintomas. No total, 106 profissionais de saúde compareceram ao trabalho enquanto estavam doentes, levando à exposição de até 234 pacientes, dos quais 137 pacientes receberam quimioprofilaxia antiviral posteriormente^21^. O motivo mais comum foi o “senso de dever profissional” ^(^ ^40^. Esses resultados convergem com estudo qualitativo realizado com enfermeiros brasileiros, no qual o compromisso profissional foi citado como motivo do presenteísmo, além da necessidade financeira^41^.

Quanto às consequências para a saúde individual e organizacional, os estudos confirmaram relações entre presenteísmo, desfechos clínicos desfavoráveis e baixa qualidade da assistência, como aumento de erros médicos e comprometimento da segurança do paciente^18^ ^-^ ^20^ ^,^ ^27^ ^,^ ^31^ ^,^ ^34^. Em um destes estudos, a origem de um surto de infecção respiratória em uma unidade de tratamento de pequeno porte para saúde mental foi atribuída ao presenteísmo de apenas um profissional de saúde. As infecções foram disseminadas por este profissional que foi trabalhar doente e infectou 17 pacientes no total. Destes, cinco desenvolveram complicações de pneumonia e um total de US$12.324 em custos médicos adicionais foi incorrido como resultado do surto^31^.

Tais resultados desfavoráveis são os custos do presenteísmo, muitas vezes invisíveis, para as instituições, pois, na maioria das vezes, as perícias realizadas pouco investigam de forma aprofundada as variáveis envolvidas na prática do presenteísmo e as consequências dele oriundas. É necessário compreender este fenômeno sob uma perspectiva sistêmica e multidimensional, pois o comprometimento da saúde do profissional (tanto o que pratica, quanto o que trabalha com ele) reflete em agravamento de doenças pré-existentes, fadiga, menor produtividade, aumento do risco de absenteísmo futuro, *burnout*, elevação de custos com afastamentos futuros, doenças crônicas e intenção de deixar o emprego^18^ ^-^ ^19^. Todas estas consequências, a nível individual, refletem em consequências para pacientes e instituições, tornando-se um ciclo vicioso de adoecimento organizacional.

As pessoas que praticam o presenteísmo podem ter benefícios de saúde a curto prazo, mas o seu bem-estar pode deteriorar-se ao longo do tempo^42^. Outros estudos relatam que o presenteísmo pode ter aspectos positivos e ser funcional em determinados contextos quando os recursos apropriados estão disponíveis^43^. No entanto, os achados desta pesquisa indicam que as consequências do presenteísmo são predominantemente negativas para a segurança do paciente.

Quanto às intervenções para reduzir o presenteísmo entre profissionais da saúde hospitalares, apenas dois estudos enfatizaram este tópico^22^ ^-^ ^23^. Estes abordaram intervenções a nível de saúde ocupacional e criação de centros para o bem-estar profissional, não havendo intervenções organizacionais específicas mais robustas. Isso é notável, visto que ambientes com forte apoio organizacional demonstram promover uma maior autoestima entre os trabalhadores e reduzir os índices de presenteísmo.

Os centros de bem-estar foram instalações criadas durante a pandemia da COVID-19 para oferecer conforto, por meio de elementos sensoriais positivos (iluminação suave, bolas antiestresse, óleos essenciais) e acesso a primeiros socorros psicológicos para profissionais de saúde no combate ao coronavírus^22^ ^-^ ^23^.

São necessários investimentos em intervenções capazes de incluir o comprometimento, o apoio mútuo e o suporte organizacional como pautas das políticas de gestão de pessoas das instituições hospitalares, os quais consigam inserir os profissionais como protagonistas nos processos de tomada de decisão, planejamento da força de trabalho e participação na discussão dos assuntos relacionados ao presenteísmo e sua relação com a qualidade da assistência.

Ademais, sugerem-se intervenções tanto em âmbito organizacional quanto individual. Entre as intervenções organizacionais, destacam-se, ainda, melhorar a distribuição de salários, equilibrar a carga de trabalho e promover recompensas baseadas no *feedback* dos funcionários. É fundamental fortalecer o suporte organizacional, criando um ambiente onde os trabalhadores se sintam valorizados, escutados e apoiados, investindo em lideranças sensíveis e empáticas, comprometidas com o bem-estar da equipe. A promoção de uma cultura organizacional que valorize os trabalhadores, juntamente com a implementação de políticas claras de licença médica e gestão da sobrecarga de trabalho, também é crucial. 

No âmbito individual, recomenda-se contemplar, nos processos seletivos, a avaliação das competências emocionais para o trabalho, que incluem autoconsciência, autocontrole, autorrealização e empatia. É importante também oferecer capacitações contínuas e apoio psicológico para fortalecer a autoestima dos profissionais. Por fim, campanhas internas de conscientização sobre os riscos do presenteísmo são essenciais para alertar os profissionais sobre os impactos negativos de trabalhar doente tanto para si quanto para as equipes e os pacientes.

Diante disso, ressalta-se a necessidade de avanços na formulação de políticas institucionais que priorizem a saúde organizacional e a promoção de ambientes de prática positivos, e que reconheçam o presenteísmo como um fenômeno que, se negligenciado, compromete tanto o cuidado prestado quanto a saúde dos próprios profissionais. Os gestores hospitalares, portanto, exercem papel fundamental na redução do presenteísmo. A realização de novos estudos, com enfoque qualitativo e longitudinal, é essencial para aprofundar a compreensão das causas e consequências do presenteísmo e embasar a construção de intervenções eficazes.

Dentre algumas limitações, alguns artigos não apresentaram informações detalhadas sobre o setor hospitalar ou da categoria profissional dos participantes. Em vários casos, os estudos se referem de modo geral a “profissionais da saúde” ou “trabalhadores hospitalares”, sem distinção de função ou área de atuação. Essa ausência de detalhamento limita a comparação entre contextos institucionais e categorias específicas dentro do ambiente hospitalar. Apesar da gravidade dos impactos, poucos estudos abordam intervenções para prevenção ou mitigação do presenteísmo. As estratégias identificadas foram pontuais, como a implementação de centros de bem-estar, mas não há consenso sobre sua efetividade. Além disso, a predominância de estudos transversais nesta revisão também revela limitação para os resultados.

## Conclusão

Esta revisão de escopo cumpriu seu caráter de mapeamento e evidenciou que o presenteísmo é um fenômeno prevalente e multifatorial entre profissionais da saúde em ambiente hospitalar. Os resultados apontam que os profissionais frequentemente comparecem ao trabalho mesmo em condições de saúde comprometidas, motivados por fatores como pressão organizacional, cultura institucional que valoriza o “trabalhar apesar da doença”, ausência de políticas de apoio, medo de represálias e sentimento de responsabilidade com os pacientes.

As consequências são amplas: na dimensão individual, o presenteísmo contribui para o agravamento de doenças crônicas, estresse, fadiga e redução da capacidade laboral; no âmbito organizacional, ele compromete a produtividade, eleva os custos diretos e indiretos com saúde e impacta negativamente o clima de segurança do paciente, com maior risco de erros e disseminação de infecções.

## Data Availability

O conjunto de dados deste artigo está disponível na página da RLAE no repositório SciELO Data, no link https://doi.org/10.17605/OSF.IO/GVNX3

# Translation: Presentismo de los profesionales de la salud en un contexto hospitalario: una revisión de alcance

**DOI:** 10.1590/1518-8345.8286.4899

**Authors:** Cynthia Lima Sampaio, Thailane Cardoso Soares, Victoria Lohreine de Carvalho Grangense, Bruna Victória da Silva Passos, Roberta Meneses Oliveira, Joselany Áfio Caetano

**Affiliations:** 1 Universidade Federal do Ceará, Departamento de Enfermagem, Fortaleza, CE, Brasil.

**Keywords:** Hospitales, Presentismo, Salud Laboral, Gestión en Salud, Personal de Salud, Revisión de Alcance

## Abstract

**Objetivo:**

mapear la prevalencia del presentismo entre los profesionales de la salud en el entorno hospitalario, identificar factores relacionados, consecuencias para la seguridad del paciente y la salud laboral, así como las intervenciones utilizadas.

**Método:**

revisión de alcance basada en las recomendaciones del JBI *Reviewer’s Manual*, incluyendo estudios disponibles íntegros en cinco bases de datos, sin restricciones de tiempo ni idioma.

**Resultados:**

los 31 artículos seleccionados mostraron una alta prevalencia de presentismo entre los profesionales de la salud en el ámbito hospitalario, y los principales factores relacionados fueron enfermedades (depresión e infecciones), aspectos ocupacionales (sobrecarga de trabajo) y aspectos organizacionales (falta de apoyo de los colegas). Las consecuencias para la salud del trabajador fueron empeoramiento de la salud, estrés, fatiga/agotamiento y deterioro de la capacidad laboral, mientras que, para la seguridad del paciente, hubo un mayor riesgo de infecciones y errores clínicos. Pocos estudios han informado sobre intervenciones, como los centros de bienestar ocupacional. Las consecuencias también impactaron aspectos económicos con pérdida de productividad y mayores costos de atención médica.

**Conclusión:**

la prevalencia de presentismo entre los profesionales de la salud en el ámbito hospitalario es alta y constituye un fenómeno multifactorial con consecuencias negativas para individuos, organizaciones y pacientes. El análisis multidimensional del presentismo puede contribuir al establecimiento de políticas y programas de salud organizacional en hospitales. Dada la escasez de estudios de intervención, es necesario avanzar en el conocimiento sobre el tema. El protocolo de investigación está contenido en la plataforma *Open Science Framework*: https://doi.org/10.17605/OSF.IO/GVNX3.

## Destacados

**(1)** El presentismo es un fenómeno prevalente y multifactorial entre los profesionales sanitarios en entornos hospitalarios. **(2)** El presentismo contribuye al agravamiento de enfermedades crónicas, el estrés, la fatiga y la reducción de la capacidad laboral. **(3)** En el contexto organizacional, compromete la productividad, aumenta los costes sanitarios directos e indirectos **(4)** Afecta negativamente a la seguridad del paciente, con un mayor riesgo de errores y propagación de infecciones. **(5)** Se necesitan estudios de intervención para avanzar en el conocimiento sobre este tema.

## Introducción

Las instituciones de salud son reconocidas como entornos laborales de alta complejidad y exposición a riesgos, abarcando aquellos comunes a otros sectores además de los inherentes a las prácticas de atención. Los profesionales sanitarios, especialmente aquellos que trabajan en hospitales, están expuestos a una amplia variedad de riesgos, ya sean biológicos, químicos, físicos, ergonómicos, además de problemas organizativos y riesgos psicosociales^1^.

En este contexto, el absentismo y el presentismo son resultados comunes de estas exposiciones y afectan a la productividad y el bienestar de los profesionales. Sin embargo, aunque el absentismo entre los profesionales de enfermería es ampliamente estudiado debido a su alto coste para los sistemas de salud, el presentismo es un fenómeno más complejo y difícil de medir, ya que implica múltiples factores^2^.

Es posible ejemplificar los altos costes del ausentismo con la pérdida anual de aproximadamente 4 millones de dólares estadounidenses para la Provincia Oriental de Arabia Saudí^2^. En relación con el presentismo, los estudios existentes muestran una gran heterogeneidad metodológica y resultados contradictorios respecto a las asociaciones con variables ocupacionales u organizativas. Esto dificulta la formulación de conclusiones definitivas en el campo de la salud^3^.

Además, el presentismo sigue siendo un concepto en construcción, pero ya se reconoce como la presencia del profesional en el trabajo incluso cuando existen limitaciones físicas y mentales que impiden su actividad laboral, aunque también existe una clasificación de presentismo no relacionada con la enfermedad (por razones personales, como el conflicto entre la vida profesional y personal, la percepción de falta de apoyo organizativo, estrés, etc.)^3^.

El escenario posterior a la pandemia de COVID-19 ha incrementado el interés en este fenómeno, ya que los trabajadores de salud se enfrentaron a condiciones excepcionales de carga emocional, física y organizativa, con posibles repercusiones duraderas en el presentismo, la seguridad del paciente y la salud laboral^4^. Estudios recientes indican una alta prevalencia de presentismo entre los profesionales hospitalarios, asociado con agotamiento y repercusiones en la calidad de la atención y la seguridad del paciente^4^ ^-^ ^5^.

A pesar de las evidencias prácticas sobre el impacto del presentismo en los servicios de salud, faltan estudios que integren, de manera más profunda, la prevalencia, factores asociados, consecuencias e intervenciones específicas en el contexto hospitalario.

El presente estudio está justificado en vista de la necesidad de investigar más sobre la relación entre el presentismo, el agotamiento físico y mental, el agotamiento, el síndrome de *burnout*, el estrés y la depresión, para permitir intervenciones eficaces.

En el campo de la enfermería, la relevancia de este estudio radica en que esta categoría representa la mayor fuerza laboral en las instituciones hospitalarias y desempeña un papel central en la atención directa al paciente. El presentismo entre los profesionales de enfermería puede tener implicaciones para la seguridad de la atención, la calidad de la atención y aumentar los costes organizativos.

Comprender el presentismo desde la perspectiva de la gestión de enfermería es esencial para apoyar intervenciones que promuevan entornos laborales más saludables, reduzcan los riesgos laborales y favorezcan prácticas de atención segura.

Por lo tanto, el objetivo de este estudio fue mapear la prevalencia del presentismo entre los profesionales de la salud en el entorno hospitalario, identificar factores relacionados, consecuencias para la seguridad del paciente y la salud laboral, así como las intervenciones utilizadas.

## Método

### Tipo de estudio

El estudio es una revisión de alcance que siguió los pasos recomendados por el JBI *Reviewer’s Manual*, desarrollado a través de cinco pasos: formulación de la pregunta de investigación; identificación de estudios relevantes; selección de estudios; extracción y análisis de datos; Síntesis y construcción del informe. Para la redacción del estudio se utilizaron los ítems preferidos de reporte para revisiones sistemáticas y metaanálisis para revisiones de alcance (PRISMA-ScR). El registro del protocolo de investigación está listado en la plataforma *Open Science Framework* (OSF) bajo el https://doi.org/10.17605/OSF.IO/GVNX3 de Identificador de Objetos Digitales (DOI). La búsqueda en las diferentes bases de datos se realizó en septiembre de 2024.

### Pregunta de investigación

La pregunta del estudio se elaboró mediante el acrónimo PCC, según las recomendaciones del JBI. La población (P) se consideraba profesional de la salud; concepto de interés (C), presentismo; y, como contexto (C), hospital; formulando la siguiente pregunta: “¿Cuál es el panorama del tema del presentismo de los profesionales de la salud en el entorno hospitalario?”

Para responder a la pregunta orientadora, se plantearon otras preguntas: P1. ¿Cuál es la prevalencia del presentismo entre los profesionales sanitarios en el entorno hospitalario? P2. ¿Cuáles son los factores que pueden influir en el presentismo en esta población?; P3. ¿Cuáles son las consecuencias del presentismo para la seguridad del paciente y la salud profesional en el entorno hospitalario?; y P4. ¿Qué intervenciones individuales y organizativas se sugieren para reducir el presentismo en el entorno hospitalario?

### Criterios de selección

La revisión abordó estudios completamente publicados que respondieron a la pregunta de investigación. Los criterios de inclusión fueron: publicaciones sin restricciones de tiempo ni idioma, con texto completo disponible. Se excluyeron editoriales, vídeos, páginas web, noticias, *preprints* y resúmenes. 

### Estrategia de búsqueda

La búsqueda se realizó en las siguientes bases de datos: MEDLINE/PubMed (a través de la Biblioteca Nacional de Medicina), *Web of Science*, CINAHL (vía EBSCO), *Scopus* y *Embase*, en los siguientes pasos: 1) búsqueda inicial en MEDLINE para identificar estudios sobre el tema y seleccionar las palabras contenidas en estas publicaciones; 2) elaboración de la estrategia de búsqueda; 3) validación de la estrategia de búsqueda desarrollada con un bibliotecario; 4) Uso de la nueva estrategia. 

La estrategia de búsqueda adoptada inicialmente fue “presenteeism” OR “sickness presence” AND “hospitals” AND “healthcare workers”. Tras consultar con la bibliotecaria, la estrategia se definió de la siguiente manera, utilizándose el MeSH *(Medical Subject Headings)*: ((Presenteeism OR “Sickness Presence” OR “Presence, Sickness”) AND (Hospitals OR “Hospital Administration”) AND (“Health Personnel” OR “Personnel, Health” OR “Healthcare Workers” OR “Healthcare Worker” OR “Health Care Providers” OR “Health Care Provider” OR “Provider, Health Care” OR “Healthcare Providers” OR “Healthcare Provider” OR “Provider, Healthcare” OR “Health Care Professionals” OR “Health Care Professional” OR “Professional, Health Care”) y de la siguiente manera, utilizándose el EMTREE: ((‘presenteism’ OR ‘sickness presence’ OR ‘working while ill’ OR ‘working while sick’ OR ‘presenteeism’) AND (‘health care practitioner’ OR ‘health care professional’ OR ‘health care provider’ OR ‘health care worker’ OR ‘health personnel’ OR ‘health profession personnel’ OR ‘health worker’ OR ‘healthcare personnel’ OR ‘healthcare practitioner’ OR ‘healthcare professional’ OR ‘healthcare provider’ OR ‘healthcare worker’ OR ‘home health aides’ OR ‘personnel, health’ OR ‘public health officer’ OR ‘health care personnel’) AND hospital).

La estrategia con descriptores catastrados en el EMTREE se utilizó en la base de datos *Embase* y la estrategia con descriptores catastrados en el MeSH se utilizó en las otras bases de datos seleccionadas. El acceso a las bases de datos se realizó a través del Portal de Publicaciones Periódicas de la Coordinación de Perfeccionamiento del Personal de Educación Superior (CAPES).

Cabe señalar que se realizó una búsqueda en las plataformas *International Prospective Register of Systematic Reviews* (PROSPERO) y OSF para verificar la existencia de revisiones sobre el objeto de estudio, pero no se identificaron.

Una nueva búsqueda en la *Biblioteca Digital Brasileira de Teses e Dissertações* (BDTD) (Biblioteca Digital Brasileña de Tesis y Disertaciones) y en los Catálogos de teses y disertaciones de CAPES (Coordinación de Mejora de Personal de Nivel Superior) se realizó en noviembre de 2025, utilizando la siguiente estrategia: *Presenteeism* AND *Hospitals* AND “*Health Personnel*”, y solo se encontró un estudio, repetido en ambos repositorios. Este estudio ya había sido contemplado en la búsqueda inicial en las bases de datos.

### Selección de estudios

Tras la búsqueda en las bases de datos, todos los registros identificados se agruparon y se subieron a la aplicación Rayyan (*Intelligent Systematic Review*)^6^. En el primer paso, se eliminaron los duplicados. Tras esta recuperación, los títulos y resúmenes fueron leídos y seleccionados por dos revisores independientes para su evaluación, aplicando los criterios de inclusión y exclusión. Los estudios potencialmente elegibles se obtuvieron en texto completo y fueron evaluados por los mismos revisores. Los desacuerdos sobre los estudios que conformarían la muestra se discutieron entre los revisores y, cuando no hubo consenso, se llamó a un tercer revisor.

Los evaluadores participantes tienen un título en Enfermería y forman parte de un Centro de Enseñanza, Investigación y Extensión en Gestión y Atención a la Salud en una universidad federal, en la línea de investigación: Atención y Gestión de la Atención a la Salud Organizacional y Seguridad del Paciente. La fase de selección y selección fue inicialmente realizada por dos revisores independientes, uno de los cuales era estudiante de doctorado con experiencia en revisión de alcance y otro miembro del programa de iniciación científica. El primer revisor seleccionó 31 estudios y el segundo, 15, con acuerdo en 12 registros, lo que corresponde a un 35% de acuerdo. En vista de la divergencia observada, un tercer revisor, un estudiante de máster con experiencia en revisión de alcance, realizó un nuevo cribado y seleccionó 29 estudios, todos coincidiendo con las elecciones del primer revisor, resultando en un acuerdo total entre ellos (kappa = 1,00).

Las razones de la exclusión en texto completo se registraron y presentaron en el diagrama de flujo PRISMA-ScR.

### Instrumento utilizado para la recopilación de información

La extracción de datos se realizó utilizando un instrumento desarrollado por los autores, basado en la forma sugerida por el manual y las publicaciones del protocolo JBI, que se adaptó al objeto de estudio^7^. Los datos extraídos fueron: identificación del estudio (autor, título, año, país, diseño, objetivo); participantes (número total, formación profesional, lugar de estudio); resultados encontrados (prevalencia del presentismo entre los trabajadores de salud en el entorno hospitalario, factores relacionados, consecuencias para la seguridad del paciente y la salud laboral, intervenciones utilizadas para reducir el presentismo); conclusiones.

Las informaciones fueron organizadas en una hoja de cálculo electrónica en Microsoft Excel^®^, lo que permitía el control de los pasos y la verificación cruzada de los datos. No se modificaron ni eliminaron datos sin acuerdo entre los revisores.

### Procesamiento y análisis de datos

A partir de los datos extraídos, se realizó un análisis descriptivo y se construyeron tablas y figuras con los datos de las publicaciones. Los resultados de la investigación y el proceso de inclusión del estudio se informan íntegramente en la revisión final de alcance y se presentan en un diagrama de flujo titulado *Preferred Reporting Items for Systematic Reviews and Meta-analyses extension for scoping review* (PRISMA-ScR). 

La síntesis de los resultados se organizó de forma narrativa, agrupando los hallazgos según similitudes conceptuales y metodológicas. Se decidió no realizar una evaluación crítica de la calidad metodológica, en línea con las recomendaciones del JBI para las revisiones de alcance.

Los factores que influyen en el presentismo se identificaron en los estudios mediante pruebas estadísticas de asociación y correlación, incluyendo: chi-cuadrado (χ²), test t de Student y ANOVA para asociaciones, y correlación (r) de Pearson y Spearman para medir correlaciones^8^.

### Aspectos éticos

El presente estudio no involucró a personas y los datos utilizados se consideran secundarios en el dominio público con acceso libre. Además, se insertaron todas las referencias relativas a derechos de autor^9^. Por lo tanto, no era necesario presentarlo al Comité de Ética en Investigación.

## Resultados

Las búsquedas dieron lugar a 216 registros. Tras la exclusión de los duplicados, quedaban 123 para la selección. En el análisis de títulos y resúmenes, se seleccionaron 56; y, tras leer los estudios completos, se incluyeron 31 en la revisión (Figura 1).

**Figura 1:** Diagrama de flujo PRISMA utilizado para la identificación y selección de estudios. Fortaleza, CE, Brasil, 2025

Fuente: Diagrama de flujo de selección del estudio PRISMA

Los 31 estudios muestran una amplia diversidad en cuanto a país de origen, año de publicación, diseño metodológico e instrumentos utilizados (Figura 2). La mayoría son estudios transversales (n = 22; 70,97%), seguidos de tres estudios longitudinales prospectivos (9,68%) y tres estudios retrospectivos (9,68%). Además, se identificaron un estudio metodológico (3,23%), una revisión sistemática (3,23%) y una carta al editor (3,23%).

**Figura 2:** Identificación de los estudios incluidos en la muestra según el código, país, año de publicación, diseño, preguntas respondidas en la revisión e instrumentos utilizados. Fortaleza, CE, Brasil, 2025

Código del artículo	País/Año	Diseño	Respuesta a las preguntas de Revisión (P1 a P4)	Instrumentos de medición
A1^10^	Suiza, 2021	Longitudinal prospectivo	P1	*Charlson Comorbidity Index* Antecedentes de la vacunación contra la gripe y características del hogar que podrían interferir con el riesgo de gripe Diarios de síntomas respiratorios y adherencia a la mascarilla
A2^11^	Nigeria, 2021	Transversal	P1 y P2	*Oldenburg Burnout Inventory* (OLBI) *Stanford Presenteeism Scale* (SPS-6) *Patient Health Questionnaire-2* (PHQ-2)
A3^12^	China, 2021	Transversal	P2	*Perceived Ability to Work Scale* (PAWS) *Challenge and Hindrance-related Self-Reported Stress Scale* (C-HSS) *Short Form Health Survey* (SF-8) *Public Service Motivation Scale*
A4^13^	China, 2019	Transversal	P1 y P2	*Perceived Ability to Work Scale* (PAWS) *Short Form Health Survey* (SF-8) *Challenge and Hindrance-related Self-Reported Stress Scale* (C-HSS) *Short-form PSM Scale* *Job in General Scale* *Turnover Intention Scale* *Job Performance Scale* *Chirurgisches Qualitätssiegel Scale*
A5^14^	China, 2022	Transversal	P1 y P2	Escala de Niehoff & Moorman (justicia distributiva); Escala de Pierce et al (autoestima organizacional) *Perceived Ability to Work Scale* (PAWS) Escala de Eisenberger, et al. (Apoyo Organizacional Percibido)
A6^15^	Turquía, 2024	Metodológico	P2	*Stanford Presenteeism Scale* (STAS) *Happiness At Work Scale* *Burnout Scale Short Version*
A7^16^	China, 2024	Transversal	P2	*Impact of Events Scale* (IES) *Turnover intention Scale* (PTSD) *Stanford presenteeism Scale* (STAS) *Social Support Scale* (SSS)
A8^17^	Brasil, 2021	Transversal	P1 y P2	*Stanford Presenteeism Scale* (STAS) - versión brasileña *Safety Attitudes Questionnaire* (SAQ)
A9^18^	Malta, 2020	Transversal	P2 y P3	Cuestionario sobre las percepciones de enfermedades durante episodios de presentismo y absentismo *Brief Illness Perception Questionnaire* (B-IPQ)
A10^19^	Francia, 2022	Revisión sistemática	P3	*Stanford Presenteeism Scale* (SPS-6) *Work Limitations Questionnaire* (WLQ) *Work Productivity and Activity Impairment* (WPAI) *Health and Work Performance Questionnaire* (HPQ) *Perceived Ability to Work Scale* (PAWS)
A11^20^	Brasil, 2022	Transversal	P1, P2 y P3	*Stanford Presenteeism Scale* (SPS-6) *Work Ability Index* (WAI)
A12^21^	Japón, 2020	Carta al editor	P1	Dos preguntas abiertas sobre síntomas, reconocimiento y la enfermedad y razones para ir al trabajo estando enfermo.
A13^22^	Reino Unido, 2024	Transversal	P4	*Warwick-Edinburgh Mental Wellbeing Scale* (WEMWBS) Cuatro medidas globales de un solo ítem de estrés laboral, satisfacción laboral, presentismo e intenciones de rotación.
A14^23^	Reino Unido, 2020	Transversal	P1 y P4	*Warwick-Edinburgh Mental Wellbeing Scale* (WEMWBS) Escalas globales de un solo ítem (estrés, satisfacción, intención de salida y presentismo); *Utrecht Work Engagement Scale* (UWES - subescala de dedicación, 3 ítens) Preguntas abiertas sobre la percepción, los beneficios y las barreras de los centros.
A15^24^	Australia, 2021	Transversal	P1 y P2	Pregunta sobre cuántas veces han ido a trabajar cuando estaban enfermos o no se sentían bien en los últimos 12 meses.
A16^25^	China, 2017	Transversal	P1 y P2	*Perceived Ability to Work Scale* (PAWS)
A17^26^	Estados Unidos, 2017	Transversal	P1	Cuestionario autorreportado en línea y anónimo
A18^27^	Portugal, 2016	Longitudinal retrospectivo	P1 y P3	*Work Limitations Questionnaire8* (WLQ8) (versión abreviada de 8 ítems) *Stanford Presenteeism Scale* (SPS6)
A19^28^	China, 2019	Transversal	P1 y P2	*Perceived Ability to Work Scale* (PAWS) *Short Form Health Survey* (SF-8) *Challenge and Hindrance-related Self-Reported Stress Scale* (C-HSS) *Short-form PSM scale* Escala de Ironson, et al. (satisfacción laboral) Escala de Singh, et al. (intención de rotación) Escala de Darwish (rendimiento laboral) *Chirurgisches Qualitätssiegel Scale*
A20^29^	Australia, 2020	Longitudinal retrospectivo	P1	Presentismo inferido por ausencia de registro de baja médica durante el periodo de infección confirmada Absentismo medido por horas de permiso registradas / horas totales programadas × 100 Datos laboratoriales, administrativos y nóminas integrados mediante número de identificación del empleado.
A21^30^	China, 2019	Transversal	P2	*Perceived Ability to Work Scale* (PAWS)
A22^31^	Japón, 2022	Longitudinal retrospectivo	P3	No se utilizó ningún cuestionario ni escala estandarizada de presentismo
A23^32^	Croacia, 2017	Transversal	P1 y P2	*Patient Safety Culture;* *Health and Work Performance Questionnaire* (HPQ)
A24^33^	China, 2018	Transversal	P1 y P2	*Perceived Ability to Work Scale* (PAWS) *Challenge and HindranceRelated SelfReported Stress Scale*
A25^34^	Eslovenia, 2020	Transversal	P1 y P3	Cuestionario desarrollado por los propios autores, con varias preguntas sobre salud, trabajo y comportamiento
A26^35^	España, 2018	Transversal	P1 y P2	*Stanford Presenteeism Scale6* (SPS-6)
A27^36^	Japón, 2020	Transversal	P1 e P2	Cuestionario preparado por el autor para variables demográficas y síntomas *Work Productivity and Activity Impairment - General Health* (WPAIGH)
A28^37^	Tailandia, 2022	Longitudinal prospectivo	P1	Registro directo por parte de los observadores durante el turno y cuestionario con autoinforme de los síntomas en los últimos siete días
A29^38^	Israel, 2024	Longitudinal prospectivo	P1	Informes de los participantes, combinados con datos de vigilancia activa
A30^39^	Irán, 2022	Transversal	P1 y P2	*Valuation of Lost Productivity Questionnaire* (VOLP), adaptado la a COVID-19 *WHO STEPwise*
A31^40^	Estados Unidos, 2019	Transversal	P1 y P2	Cuestionario anónimo desarrollado por los autores con los artículos cerrados sobre las razones del presentismo

En cuanto a los países, predominaron estudios realizados en China (n = 8; 25,81%), seguidos de Japón (n = 3; 9,68%), Australia (n = 2; 6,46%), Brasil (n = 2; 6,45%), Reino Unido (n = 2; 6,45%), Estados Unidos (n = 2; 6,45%) y un estudio para Suiza, Nigeria, Malta, Turquía, Francia, Portugal, Croacia, Eslovenia, España, Tailandia, Israel e Irán (3,23%). En cuanto al año de publicación, la mayor concentración se observó en 2020 (n = 6; 19,35%) y 2022 (n = 6; 19,35%), seguida de 2021 (n = 5; 16,13%), 2019 (n = 4; 12,90%) y 2024 (n = 4; 12,90%). Los otros años tuvieron menor frecuencia: 2017 (n = 3; 9,68%), 2018 (n = 2; 6,45%) y 2016 (n = 1; 3,23%).

Los instrumentos utilizados para evaluar el presentismo incluyeron escalas estandarizadas, como la *Perceived Ability to Work Scale* (PAWS) (n= 8), la *Stanford Presenteeism Scale* (SPS-6) (n= 7), el *Work Limitations Questionnaire* (WLQ) (n= 2), la *Work Productivity and Activity Impair*ment (WPAI) (n= 2), el *Health and Work Performance Questionnaire* (HPQ) (HPQ) (n= 2) y el *Valuation of Lost Productivity Questionnaire* (VOLP) (VOLP), adaptado para COVID-19 (n=1). Además, algunos estudios aplicaron sus propios cuestionarios con preguntas directas sobre la frecuencia del comportamiento presentista y otros instrumentos para medir los constructos asociados, como *Burnout*, Motivación para el trabajo, Felicidad en el trabajo, Apoyo Social, Estrés por el cambio, *Turnover*, entre otros (n= 26).

De los treinta y un artículos seleccionados para la muestra, veintitrés respondieron a la pregunta sobre el resultado de la medición del presentismo (P1), como se muestra en la Figura 3. Los estudios analizan el presentismo desde diferentes perspectivas, como trabajar enfermo o pérdida de productividad, utilizando distintas escalas. Algunos estudios proporcionan datos sobre la prevalencia del presentismo y la intensidad con la que ocurre. Otros solo revelan la prevalencia, la media o la unidad de medida (pérdida de productividad, grado o valor financiero).

**Figura 3:** Estudios sobre la prevalencia del presentismo entre profesionales en el contexto hospitalario, por país, año, número de participantes, instrumentos utilizados y principales resultados. Fortaleza, CE, Brasil, 2025

*Stanford Presenteeism Scale* (SPS-6)			
País, año	N.º de participantes	Resultados sobre presentismo	
		Prevalencia	Media
Nigeria, 2021^11^	155	no informado	22,1 (DE* 5,1)
España, 2018^35^	323	52,9%	19,84 (DE* 4,2)
Brasil, 2021^17^	754	43,5%	20,9 (DE* 4,7)
Brasil, 2022^20^ ^)^	299	65,6%	21,36 (DE* 4,7)
***Perceived Ability to Work Scale* (PAWS)**			
China, 2017^25^	1392	no informada	7,25 (DE* 1,79) a 7,63 (DE* 1,57)
China, 2019^13^	572	no informada	3,92 (DE* 2,33) a 4,12 (DE* 2,38)
China, 2019^28^	1434	no informada	7,44 (DE* 1,716) a 7,71 (DE* 1,59)
China, 2018^33^	1392	no informada	2,37 (DE* 1,67) a 2,75 (DE* 1,79)
China, 2022^14^	1122	no informada	2,09 (DE* 1,46) a 2,35 (DE* 1,61)
***Work Limitations Questionnaire- 8* (WLQ-8) e SPS-6**			
Portugal, 2016^27^	188	no informada	WLQ-8: Pérdida de 19,56% de productividad por día laborable. SPS-6: enfermeros 1,95 (0,96) y asistentes 2,28 (0,70)
***Work Productivity and Activity Impairment-General Health* (WPAI-GH)**			
Japón, 2020^36^	668	62,1%	El grado de presentismo era 30
***Valuation of Lost Productivity Questionnaire* (VOLP) adaptado a la COVID-19**			
Irán, 2022^39^	250	100%	US$ 1.489,05 (DP* = US$ 1.143,15) en el periodo de tres meses.
***Health and Work Performance Questionnaire* (WHO-HPQ)**			
Croacia, 2017^32^	595	70%	6,82%
**Preguntas sobre la percepción del presentismo elaboradas por el autor**			
Japón, 2020^21^	90	Prevalencia de 48,9%	
Reino Unido, 2020^23^	819	Prevalencia de 68,6%	
Australia, 2021^24^	320	Prevalencia de 87,8%	
Estados Unidos, 2017^26^	286	Prevalencia de 92,0%	
Israel, 2024^38^	2505	Prevalencia de 72,3% (IC^†^ de 95%: 60,4-86,4).	
Eslovenia, 2020^34^	5.865	Prevalencia de 57,0%	
Tailandia, 2022^37^	240	Prevalencia de 23,0%	
**Análisis de casos de gripe confirmados en laboratorio**			
Australia, 2020^29^	1.540	Prevalencia de 14,1%	
Suiza, 2021^10^	152	Prevalencia de 67,9%	
**Otras escalas**			
Japón, 2020^40^	14	Prevalencia de 64,0%	

*DP = Desviación estándar; ^†^IC = Intervalo de confianza

Los resultados muestran una amplia variación en la prevalencia del presentismo, con oscilaciones del 14,1% en Australia al 100% en Irán. En cuanto a las medias de las escalas, se observaron variaciones de 2,37 (DE = 1,67) a 7,71 (DE = 1,59) en el PAWS, y de 19,84 (DE = 4,2) a 22,1 (DE = 5,1) en el SPS-6.

Solo tres estudios compararon el presentismo entre diferentes categorías profesionales. En uno de ellos, las enfermeras tenían un nivel de presentismo más bajo (14,48%) en comparación con los asistentes operativos (27,19%)^27^. En otro, el presentismo era mayor entre los médicos^39^. Por otro lado, un estudio brasileño encontró una menor pérdida de productividad entre los médicos en comparación con todos los profesionales de la muestra (23,85 puntos), seguidos de los técnicos de enfermería y enfermeros, que obtuvieron puntuaciones de 20,91 y 20,65 puntos, respectivamente, indicando una menor reducción en su productividad ante problemas de salud^20^.

Diecinueve artículos respondieron sobre los factores que pueden influir en el presentismo (P2). Estos datos se encontraron en los artículos mediante asociaciones y correlaciones (Figura 4). Un estudio con una pregunta directa sobre el motivo de trabajar enfermo obtuvo un porcentaje mayor (56%) de respuestas para “sentido del deber como profesional de la salud”^40^.

**Figura 4:** Factores que pueden influir el presentismo verificados a través de asociaciones y correlaciones en los estudios revisados. Fortaleza, CE, Brasil, 2025

Se encontraron asociaciones significativas entre el presentismo y los factores identificados en los estudios revisados
**Factores demográficos:** 🚬Tabaquismo (Fumadores con presentismo más alto: OR* = 2,99, p = 0,030)^(39)^ 👴Edad (Edad media: OR* = 11.189, p = 0,046)^(39)^ 👴Edad (30 años o menos, p = 0,023)^(15)^ 💍Soltero (p = 0,014)^(15)^ 🏘Vivir solo (p = 0,030)^(15)^ 🚻Género (Mujeres con un riesgo 1,88 veces mayor, IC^†^: 1,06-3,32, p = 0,03)^(20)^ 🚻Género (Hombres) (p < 0,05)^(13)^
**Factores de salud y enfermedades:** 🤯Estrés (p < 0,01)^(35)^ 😔Depresión (p < 0,015)^(11)^ 💪Trastornos musculoesqueléticos (p < 0,05)^(18)^ ❤ Enfermedades crónicas (p < 0,05)^(18)^ 🤕Dolores de cabeza/migrañas (p < 0,05)^(18)^ 🦠COVID-19 (una infección: OR* = 6.816, p = 0,000), dos infecciones: OR* = 24.023, p = 0,001)^(39)^
**Factores ocupacionales:** 🏥Urgencias hospitalarias (p = 0,004)^(35)^ 📊Clima de seguridad negativo (p < 0,05)^(17)^ 💊Cultura de seguridad del paciente^(32)^ 📈Carga y duración de la carrera (Profesionales en la mitad/final de carrera)^(13)^ 📈Trabajar 9 horas o más diarias (p < 0,001)^(15)^ 📈5 años o menos en la profesión (p = 0,012)^(15)^ 💰 Ingresos (Tercer y cuarto cuarteles con el presentismo más alto)^(39)^ 🩺Médico (p = 0,005)^(15)^ ⚖️Equidad en la distribución de recompensas y reconocimientos (por ejemplo, salarios, carga de trabajo, ascensos) presentan niveles más altos de presentismo^(14)^
**Correlaciones significativas encontradas entre el presentismo y los factores identificados en los estudios revisados**
**Carga de trabajo** Agotamiento (-0,350, p < 0,001)^‡^ ^(11)^ Desvinculación (-0,475, p < 0,001)^‡^ ^(11)^ Horas de trabajo excesivas (r = 0,105, p = 0,016)^§^ ^(36)^ Trabajo por turnos (r = 0,106, p = 0,015)^§^ ^(36)^ *Workaholism* (r = 0,232, p < 0,001)^§^ ^(36)^
**Compromiso organizacional** Compromiso organizacional (β = − 0,42, p < 0,001)^‡^ ^(28)^ Apoyo de compañeros de trabajo (β = − 0,15, p < 0,001)^‡(^ ^(28)^ ^)^ Motivación en el servicio público (β = − 0,35; p < 0,001)^‡^ ^(30)^ e (β = −0,13, p < 0,01)^‡^ ^(12)^ Compromiso afectivo (β = −0,27; p < 0,001)^‡^ ^(25)^ Felicidad en el trabajo (r = -0,567, p < 0,001)^‡^ ^(15)^
**Salud y enfermidad** Estrés de desafío (β = 0,07, p < 0,01)^§^ ^(12)^ Estrés de desafío (-0,05, p = 0,001)^‡^ ^(33)^ Estrés de impedimento (β = 0,13, p < 0,01)^§^ ^(12)^ y (0,25, p = 0,001)^§^ ^33^ Salud (β = −0,12, p < 0,01)^‡^ ^(12)^ y (r = -0,35)^‡^ ^(33)^ Estrés de desafío y estrés de impedimento (r = 0,20, r = 0,32)^§^ ^(25)^ TEPT^||^ (β = 0,638, p < 0,001)^§^ ^(16)^ Intensidad del dolor lumbar (r = 0,248, p < 0,001)^§^ ^(36)^, Duración del sueño (-0,131, p = 0,003)^‡^ ^(36)^ Síntomas depresivos (r = 0,269, p < 0,001)^§^ ^(36)^ Estabilidad emocional (r = -0,226, p ≤ 0,01)^‡^ ^(24)^ Estrés relacionado con el trabajo (r= 0,218, p ≤ 0,001)^§^ ^(24)^ *Burnout* (r= 0,653, p < 0,001)^§^ ^(15)^

*OR = *Odds Ratio*; ^†^IC = Intervalo de confianza; ^‡^Correlación negativa; ^§^Correlación positiva; ^||^TEPT = Trastorno del estrés postraumático

Los factores de asociación identificados se dividieron en: demografía, salud y enfermedades y ocupacional, lo que revela la importancia de la edad, el sexo, el tabaquismo, las enfermedades físicas y mentales, los ingresos, el sector, la carga de trabajo y el clima de seguridad.

Se observó que la presencia de factores como agotamiento, desconexión, compromiso organizativo, apoyo de compañeros, motivación en el servicio público, compromiso afectivo, felicidad en el trabajo, mejor salud general, mayor duración del sueño y estabilidad emocional se correlacionaron con un menor presentismo.

Por otro lado, una mayor carga de trabajo, el trabajo por turnos, el estrés por desafíos e impedimentos, los síntomas depresivos, una mayor intensidad de dolor lumbar y la presencia de síntomas de TEPT (Trastorno del estrés postraumático) se correlacionaron con un mayor presentismo.

En cuanto a las consecuencias del presentismo (P3), se encontraron siete artículos que respondían sobre las repercusiones para la seguridad de pacientes y trabajadores, así como sobre el impacto en la capacidad laboral y económica, como se muestra en la Figura 5.

**Figura 5:** Mapa mental sobre las consecuencias del presentismo. Fortaleza, CE, Brasil, 2025

En cuanto a las intervenciones individuales y organizativas adoptadas (P4), solo dos estudios informaron del uso de centros de bienestar como estrategia institucional. En una de estas investigaciones, se observó que los profesionales de la salud sin acceso a centros de bienestar presentaban niveles más altos de presentismo (β = −0,22; p < 0,001) y eran, de media, más jóvenes (β = 0,09; p = 0,002). Además, se identificó una interacción significativa entre el presentismo y el acceso al centro de bienestar en los niveles de bienestar de los profesionales, con F(1,791) = 18,65; p < 0,001; ηp² = 0,02. Los profesionales que presentaron presentismo y accedieron al centro informaron niveles más altos de bienestar (media = 3,30; desviación estándar - DE = 0,04), en comparación con quienes accedieron al centro, pero no reportaron presentismo (media = 3,06; SD = 0,040)^22^.

Por otro lado, un segundo estudio no identificó diferencias estadísticamente significativas entre los profesionales que acudieron a los centros de bienestar y aquellos que no accedieron a los centros de bienestar respecto a las variables: percepción del estrés en el trabajo, satisfacción laboral, presentismo o intención de rotación^23^.

## Discusión

Los resultados presentados nos permiten visualizar una visión completa de varios aspectos del presentismo. Las preguntas orientadoras sobre la prevalencia del presentismo y los factores que lo influyen se respondieron en gran medida a través del mapeo realizado. Sin embargo, en pocos estudios se han encontrado respuestas a preguntas sobre consecuencias e intervenciones.

Los hallazgos refuerzan la heterogeneidad de los instrumentos para medir el presentismo y la importancia de considerar múltiples factores contextuales y metodológicos al interpretar los resultados. En cuanto a la prevalencia del presentismo evaluado, los resultados varían según el instrumento de medición utilizado.

La PAWS fue la escala más utilizada para investigaciones sobre la pérdida de productividad, seguida por la SPS-6. El instrumento original considera la autopercepción de la capacidad laboral, con resultados que van de 0 a 10, en los que valores más altos indican mayor capacidad de trabajo y mejor rendimiento. En los estudios incluidos en esta revisión, los autores invierten las puntuaciones para una interpretación más intuitiva, en la que puntuaciones más altas representan un mayor presentismo, mientras que las puntuaciones más bajas reflejan un menor presentismo o una mayor percepción para trabajar. El SPS-6, por otro lado, permite medir la influencia de los problemas de salud en la calidad del trabajo y en el rendimiento del profesional, demostrando ser un instrumento fácil de entender, que favorece su finalización y análisis^15^.

Hay una concentración de estudios más recientes, principalmente entre 2017 y 2023. Esto puede estar relacionado con el creciente interés en el tema, especialmente tras la pandemia de COVID-19, que intensificó los debates sobre la salud laboral y el absentismo. Sin embargo, incluso antes de la pandemia, las tasas de presentismo ya eran altas en algunos contextos^27^.

La prevalencia del presentismo varió considerablemente, desde el 14,1% en Australia^29^ hasta el 100% en Irán^39^, con muchos estudios mostrando tasas superiores al 50%. Cabe destacar que países como Australia (87,8%)^24^, Israel (72,3%)^38^, Reino Unido^23^ y Estados Unidos^26^ (con variaciones entre el 68,6% y el 92%) presentaban una prevalencia significativamente alta de presentismo, lo que puede reflejar una mayor presión institucional para asistir al trabajo incluso en condiciones de salud inadecuadas, o incluso indicar la presencia de culturas organizativas más permisivas y tolerantes con esta práctica.

En cuanto al origen del presentismo, los resultados sugieren la influencia de factores individuales, como el compromiso profesional y el miedo a represalias, y factores organizativos, como la alta carga de trabajo y los bajos niveles de personal. Estos factores merecen una interpretación cuidadosa, ya que las correlaciones encontradas entre variables predictoras y el resultado del presentismo pueden ser ambivalentes. Por ejemplo, en un estudio, el agotamiento y el desinterés se correlacionaron con un índice de presentismo más bajo^11^, mientras que en otro, los niveles de agotamiento se correlacionaron con un presentismo más alto^15^.

De manera similar, la estabilidad emocional se asoció con un aumento del presentismo^24^. Esto se puede explicar por el hecho de que los trabajadores con comportamientos de ánimo más estables tienden a presentarse a trabajar incluso cuando están enfermos.

Los resultados indican que un mayor presentismo también se asoció con una cultura organizativa desfavorable. Por otro lado, factores como el compromiso organizativo (β = − 0,42, p < 0,001), el apoyo de los compañeros (β = − 0,15, p < 0,001)^28^, la motivación en el servicio público (β = − 0,35; p < 0,001)^30^ ^)^ y (β = −0,13, p < 0,01)^12^ ^)^ y el compromiso afectivo (β = −0,27; p < 0,001) reducen el riesgo de presentismo. Además, el 75% de los profesionales informó que la causa principal de este episodio fue de naturaleza organizativa^18^.

La carga de trabajo también se ha asociado fuertemente con el presentismo. Entre los factores mencionados en los artículos se encuentran el exceso de horas de trabajo (r=0,105, p=0,016)^36^, el trabajo por turnos (r=0,106, p=0,015)^36^ ^)^ y el adicto al trabajo (r=0,232, p<0,001)^36^.

Durante la pandemia, una revisión bibliográfica identificó factores que influyeron en el presentismo, incluyendo cuestiones directamente relacionadas con la salud mental, factores individuales, factores relacionados con la situación causada por la pandemia de COVID-19 y condiciones laborales^4^.

Los factores demográficos, como la edad y el género, fueron divergentes en los estudios analizados, sin permitir generalizaciones. Al ser factores de naturaleza individual, merecen una atención especial en cada contexto analizado, teniendo en cuenta las diferencias culturales y socioeconómicas de cada región.

Las razones más frecuentemente citadas para el presentismo incluyeron síntomas leves (36/90; 40,0%) y dificultad para encontrar proveedores sanitarios de reemplazo que cubrirlos (25/90; 27,8%). Dos enfermeras afirmaron que sus supervisores les prohibieron tomar baja por enfermedad, aunque presentaban síntomas. En total, 106 profesionales sanitarios asistieron al trabajo mientras estaban enfermos, lo que llevó a la exposición de hasta 234 pacientes, de los cuales 137 recibieron quimioprofilaxis antiviral posteriormente^21^. La razón más común era el “sentido del deber profesional”^40^. Estos resultados convergen con un estudio cualitativo realizado con enfermeras brasileñas, en el que se citó el compromiso profesional como motivo del presentismo, además de la necesidad financiera^41^.

En cuanto a las consecuencias para la salud individual y organizacional, los estudios han confirmado relaciones entre el presentismo, resultados clínicos desfavorables y baja calidad de atención, como el aumento de errores médicos y la seguridad del paciente comprometida^18^ ^-^ ^20^ ^,^ ^27^ ^,^ ^31^ ^,^ ^34^. En uno de estos estudios, el origen de un brote de infección respiratoria en una pequeña unidad de tratamiento de salud mental se atribuyó al presentismo de un solo profesional de la salud. Las infecciones se propagaron gracias a este profesional, que fue a trabajar enfermo e infectó a 17 pacientes en total. De estos, cinco desarrollaron complicaciones por neumonía y un total de 12.324 dólares estadounidenses en costes médicos adicionales se incurrieron como resultado del brote^31^.

Estos resultados desfavorables son los costes del presentismo, a menudo invisibles, para las instituciones, ya que, en la mayoría de los casos, los exámenes forenses realizados no investigan en profundidad las variables implicadas en la práctica del presentismo ni las consecuencias que se derivan de él. Es necesario entender este fenómeno desde una perspectiva sistémica y multidimensional, ya que el deterioro de la salud del profesional (tanto de quienes ejercen como de quienes trabajan con él) se refleja en la agravación de enfermedades preexistentes, fatiga, menor productividad, mayor riesgo de ausentismo futuro, agotamiento, aumento de costes con futuros permisos, enfermedades crónicas y la intención de dejar el trabajo^18^ ^-^ ^19^. Todas estas consecuencias, a nivel individual, se reflejan en consecuencias para pacientes e instituciones, convirtiéndose en un círculo vicioso de enfermedad organizacional.

Las personas que practican el presentismo pueden tener beneficios para la salud a corto plazo, pero su bienestar puede deteriorarse con el tiempo^42^. Otros estudios informan que el presentismo puede tener aspectos positivos y ser funcional en ciertos contextos cuando disponen de los recursos adecuados^43^. Sin embargo, los hallazgos de esta investigación indican que las consecuencias del presentismo son predominantemente negativas para la seguridad del paciente.

En cuanto a las intervenciones para reducir el presentismo entre los profesionales sanitarios hospitalarios, solo dos estudios enfatizaron este tema^22^ ^-^ ^23^. Estas abordaron intervenciones en salud laboral y la creación de centros de bienestar profesional, y no hubo intervenciones organizativas específicas más sólidas. Esto es notable, dado que se ha demostrado que entornos con fuerte apoyo organizativo promueven una mayor autoestima entre los trabajadores y reducen las tasas de presentismo.

Los centros de bienestar fueron instalaciones creadas durante la pandemia de COVID-19 para ofrecer confort, mediante elementos sensoriales positivos (iluminación suave, pelotas antiestrés, aceites esenciales) y acceso a primeros auxilios psicológicos para los profesionales sanitarios en la lucha contra el coronavirus^22^ ^-^ ^23^.

Se necesitan inversiones en intervenciones capaces de incluir compromiso, apoyo mutuo y soporte organizacional como directrices para las políticas de gestión de personas de las instituciones hospitalarias, que pueden insertar a profesionales como protagonistas en los procesos de toma de decisiones, planificación de la plantilla y participación en la discusión de cuestiones relacionadas con el presentismo y su relación con la calidad de la atención.

Además, se sugieren intervenciones tanto a nivel organizativo como individual. Las intervenciones organizativas también incluyen mejorar la distribución de los salarios, equilibrar la carga de trabajo y promover recompensas basadas en el *feedback* de los empleados. Es esencial fortalecer el apoyo organizativo, creando un entorno donde los trabajadores se sientan valorados, escuchados y apoyados, invirtiendo en líderes sensibles y empáticos, comprometidos con el bienestar del equipo. Promover una cultura organizativa que valore a los trabajadores, junto con la implementación de políticas claras de baja por enfermedad y la gestión de la sobrecarga de trabajo, también es fundamental.

A nivel individual, se recomienda contemplar, en los procesos de selección, la evaluación de las competencias emocionales para el trabajo, que incluyen autoconciencia, autocontrol, autorrealización y empatía. También es importante ofrecer formación continua y apoyo psicológico para fortalecer la autoestima de los profesionales. Por último, las campañas internas de concienciación sobre los riesgos del presentismo son esenciales para alertar a los profesionales sobre los impactos negativos de trabajar enfermos tanto para ellos mismos como para los equipos y los pacientes.

En vista de ello, se destaca la necesidad de avances en la formulación de políticas institucionales que prioricen la salud organizacional y la promoción de entornos de práctica positivos, y que reconozcan el presentismo como un fenómeno que, si se descuida, compromete tanto la atención proporcionada como la salud de los propios profesionales. Por tanto, los gestores hospitalarios desempeñan un papel fundamental en la reducción del presentismo. Realizar nuevos estudios, con un enfoque cualitativo y longitudinal, es esencial para profundizar en la comprensión de las causas y consecuencias del presentismo y para apoyar la construcción de intervenciones efectivas.

Entre algunas limitaciones, algunos artículos no presentaron información detallada sobre el sector hospitalario ni sobre la categoría profesional de los participantes. En varios casos, los estudios se refieren generalmente a “profesionales de la salud” o “trabajadores hospitalarios”, sin distinción de función o área de actividad. Esta falta de detalle limita la comparación entre contextos institucionales y categorías específicas dentro del entorno hospitalario. A pesar de la gravedad de los impactos, pocos estudios abordan intervenciones para prevenir o mitigar el presentismo. Las estrategias identificadas fueron puntuales, como la implantación de centros de bienestar, pero no hay consenso sobre su efectividad. Además, la predominancia de estudios transversales en esta revisión también revela limitaciones en los resultados.

## Conclusión

Esta revisión de alcance cumplió su carácter cartográfico y mostró que el presentismo es un fenómeno prevalente y multifactorial entre los profesionales de la salud en un entorno hospitalario. Los resultados indican que los profesionales suelen acudir al trabajo incluso en condiciones de salud comprometidas, motivados por factores como la presión organizativa, la cultura institucional que valora el “trabajar a pesar de la enfermedad”, la ausencia de políticas de apoyo, el miedo a represalias y el sentimiento de responsabilidad con los pacientes.

Las consecuencias son amplias: en la dimensión individual, el presentismo contribuye al agravamiento de enfermedades crónicas, estrés, fatiga y reducción de la capacidad laboral; a nivel organizativo, compromete la productividad, incrementa los costes sanitarios directos e indirectos y afecta negativamente al clima de seguridad del paciente, con un mayor riesgo de errores y propagación de infecciones.

## Data Availability

El conjunto de datos de este artículo está disponible en la página de la RLAE en el repositorio SciELO Data, en el enlace https://doi.org/10.17605/OSF.IO/GVNX3
